# MASCOT-Skyline integrates population and migration dynamics to enhance phylogeographic reconstructions

**DOI:** 10.1371/journal.pcbi.1013421

**Published:** 2025-09-26

**Authors:** Nicola F. Müller, Remco R. Bouckaert, Chieh-Hsi Wu, Trevor Bedford

**Affiliations:** 1 Division of HIV, ID and Global Medicine, University of California San Francisco, San Francisco, California, United States of America; 2 Vaccine and Infectious Disease Division, Fred Hutchinson Cancer Center, Seattle, Washington, United States of America; 3 Center for Computational Evolution, The University of Auckland, Auckland, New Zealand; 4 School of Mathematical Sciences, University of Southampton, Southampton, United Kingdom; 5 Howard Hughes Medical Institute, Seattle, Washington, United States of America; Sorbonne University, FRANCE

## Abstract

The spread of infectious diseases is shaped by spatial and temporal aspects, such as host population structure or changes in the transmission rate or number of infected individuals over time. These spatiotemporal dynamics are imprinted in the genomes of pathogens and can be recovered from those genomes using phylodynamics methods. However, phylodynamic methods typically quantify either the temporal or spatial transmission dynamics, which leads to unclear biases, as one can potentially not be inferred without the other. Here, we address this challenge by introducing a structured coalescent skyline approach, MASCOT-Skyline, that allows us to jointly infer spatial and temporal transmission dynamics of infectious diseases using Markov chain Monte Carlo inference. To do so, we model the effective population size dynamics in different locations using a non-parametric function, allowing us to approximate a range of population size dynamics. We show, using a range of different viral outbreak datasets, potential issues with phylogeographic methods. We then use these viral datasets to motivate simulations of outbreaks that illuminate the nature of biases present in the different phylogeographic methods. We show that spatial and temporal dynamics should be modeled jointly, even if one seeks to recover just one of the two. Further, we showcase conditions under which we can expect phylogeographic analyses to be biased, particularly different subsampling approaches, as well as provide recommendations on when we can expect them to perform well. We implemented MASCOT-Skyline as part of the open-source software package MASCOT for the Bayesian phylodynamics platform BEAST2.

## Author summary

Phylogeographic models allow us to track the spread of infectious diseases between different geographic areas from pathogen genome sequence data. Phylogeographic tools have to make several simplifying assumptions about the spatial spread unfolding. We here investigate how these assumptions bias the reconstruction of spatial transmission dynamics by not explicitly modeling the outbreak dynamics in the different locations. To address this challenge, we introduce a novel phylogeographic method, MASCOT-Skyline. MASCOT-Skyline is a structured coalescent approach that allows us to jointly model the time-varying outbreak dynamics as a non-parametric model in different locations together with the spatial transmission dynamics. We implemented MASCOT-Skyline as part of the Bayesian phylogenetic software platform BEAST2. Using simulations and real data examples, we explore how modeling the outbreak dynamics in different locations is crucial to accurately reconstructing the rates of movement and the location of pathogen lineages in the past.

## Introduction

Infectious diseases are a major burden on public health systems around the world [1]. Different data sources and methods exist to understand how these diseases spread quantitatively. Mainly, this relies on case data, that is, counts of when and where cases of a particular disease occurred. However, given case counts suffer from various limitations, including under-ascertainment, delays in reporting, and changes in the rate of under-ascertainment over time and between locations [2], there is continued interest in alternative data sources.

One such data source, genomic data, is increasingly being collected for infectious disease surveillance [3], though substantial differences in genomic surveillance exist across the globe [4]. Genomic data can be obtained by sequencing a subset of laboratory-confirmed cases. Pathogen genomes can give us a window into how diseases spread. Over time, random mutations to their genomes accrue. While pathogens are transmitted between individuals, the mutations leave a footprint of the transmission history in the genomes of pathogens. We can then use the pathogen genomes to reconstruct the relatedness of viruses sequenced from individuals. The evolutionary relationship of the pathogens can be approximated using a phylogenetic tree that links these individuals. From this phylogenetic tree, one can infer the transmission dynamics of infectious diseases using phylodynamic methods even if only a subset of individuals in the transmission history is sequenced [5]. Phylodynamic methods utilize the branching patterns of timed phylogenetic trees to learn about the underlying population dynamics that created them [6,7]. This information can be inferred using forward-in-time birth-death [8] or backward-in-time coalescent models [9]. Birth-death models describe how lineages multiply (birth), go extinct (death), and are sampled. The birth and death rates and their changes over time can be used to describe the transmission rates, becoming uninfectious rates, or effective reproduction numbers [10]. Coalescent models, however, describe how lineages coalesce in the past, meaning when they share a common ancestor. The rates at which two random lineages and a population share a common ancestor are lower if the population is larger and vice versa. The coalescent is typically parameterized by the effective population size (*Ne*), which is proportional to the number of infected individuals in a population and inversely proportional to the transmission rate in that population [11,12]. In contrast to case-based inference methods and birth-death methods, coalescent approaches infer population size dynamics from the relatedness of sampled genomes instead of the dynamics in the number of samples. Nonetheless, they can still suffer somewhat from biases under specific sampling assumptions [13].

Coalescent approaches can be used to model changes in pathogen prevalence or generation time, by modeling changes in the effective population size over time *Ne*(*t*). One can use deterministic parametric approaches to model changes in the population sizes over time [11] or simulate population trajectories from stochastic compartmental models [14]. Alternatively, non-parametric approaches, typically called skyline models, can be used [15]. These methods allow the effective population sizes to vary over time in a piecewise, constant fashion. Different skyline approaches vary in how changes in effective population sizes are parameterized. Some a priori assume the number of change points to be fixed, which allows the effective population size to change at coalescent events [16–18]. Other methods, typically called skygrid methods, allow the effective population sizes to vary at pre-determined points in time [19] or split the height of the tree into equally sized epochs [18]. Coalescent models have been previously deployed to, for example, study the change in the prevalence of hepatitis C [20], seasonal influenza [21], and tuberculosis [22].

An advantage of inferring transmission dynamics from genomic data is that we can learn about how cases between locations are connected. We can use this information to infer spatial transmission dynamics, which are not readily accessible from occurrence data alone. A small set of examples for this work includes studies on the early spread of HIV [23,24], the global circulation of seasonal influenza [25], and the cross-species transmission of MERS coronaviruses [26] or yellow fever [27]. Relatedly, “who infected whom” approaches can be used to determine transmission directionality between individuals (see, for example, [28]), showing the broad range of applications of methods that model population structure.

Different methods exist to reconstruct population structure, including discrete trait analyses (DTA) [29], structured birth-death [31–33], or structured coalescent methods [34–36].

Structured coalescent models model how lineages share a common ancestor within and move between sub-populations, from present to past, backward in time. The structured coalescent is parameterized by effective population size (*Ne*) and migration rates, which can be related to epidemiologically more meaningful parameters, such as the prevalence and transmission rates [12]. Structured coalescent methods largely assume that the rates of coalescence and migration are constant over time, though deterministic approaches to model parametric dynamics from compartmental models exist [37]. While structured coalescent approaches are historically not used as frequently as discrete trait analyses, there are some distinct advantages to these types of methods, including potentially being less subject to sampling biases [38]. One of the limiting factors of structured coalescent methods is their assumption of populations to be constant over time. This assumption is, however, rarely appropriate and can lead to the biased reconstruction of the within-deme and the between-deme dynamics [39].

DTA is conceptually different from structured birth-death and coalescent models. DTA only models the movement of viral lineages without explicitly modeling anything about branching processes. DTA models how lineages move conditional on a tree and is itself not a tree-generating process. In other words, one can not simulate a tree under a DTA model. They are, therefore, also referred to as neutral trait models, meaning that they model the evolution of a trait, such as geographic location, on top of an existing phylogenetic tree. DTA has arguably been the most popular of the methods described here, partly due to its ease of use and computational speed. However, biased sampling in DTA models can often lead to biased model results [38].

Here, we introduce a phylodynamic framework to infer non-parametric effective population size (*Ne*) dynamics under the marginal approximation of the structured coalescent MASCOT [40]. MASCOT computes the probability of observing a phylogenetic tree given a set of migration rates and effective population sizes by marginalizing over all potential migration histories of viral lineages. To do so, it solves a set of ordinary differential equations along the phylogenetic tree that describe the probability of any lineage *i* being in any possible discrete state *s*. MASCOT assumes that the movement of lineage *i* only depends on how much probability mass is in each state (i.e., how many lineages are on average in each state), rather than any pairwise interactions between individual lineage *i* and *j*. The effective population sizes are estimated at predefined points in time, between which we assume exponential growth dynamics [41]. As such, we allow the *Ne*’s to continuously change over time instead of assuming piecewise constant dynamics, as is typically used in skyline approaches (for example [19]). We use a Gaussian Markov Random Field (GMRF), as in [19] for unstructured populations, to model the temporal correlation between *Ne*’s. We then estimate *Ne* trajectories for each sub-population in the model using Markov chain Monte Carlo (MCMC) by using MCMC operations that learn the correlation structure between the different parameters [42]. We first show, using simulations, that we can retrieve non-parametric population dynamics and migration rates of different sub-populations from phylogenetic trees. We then show how accounting for population structure improves the inference of population dynamics and vice versa. Lastly, we compare the ancestral state reconstruction and inference results of migration rates between MASCOT-Skyline and DTA [29] using a dataset of SARS-CoV-2 sequences [30] and Susceptible-Infected-Recovered (SIR) simulations. We implemented MASCOT-Skyline as part of MASCOT [40], a package for the Bayesian phylogenetics software platform BEAST2 [43].

## Results

### Nonparametric population dynamics and migration patterns can be recovered from phylogenetic trees

We first performed a well-calibrated simulation study using a two-state structured coalescent model in MASTER [44], to validate the ability of MASCOT-Skyline to retrieve non-parametric population size dynamics. We simulated effective population size trajectories from a Gaussian Markov random field (GMRF). We sampled the natural logarithm of the effective population size at time *t* = 0 in state *a ln*(*Ne*_*a*_(*t* = 0)) from a normal distribution 𝒩(0,1). For each *Ne* at time *n* > 0, we sampled the *Ne* from ln(Ne(t=n))∼𝒩(ln(Ne(t=n−1)),0.5). Between adjacent *Ne*’s, we assume exponential growth. We repeated this to get the *Ne* trajectories of both states. We then sample the forward-in-time migration rates between the two states from an exponential distribution with a mean of 1. We compute the backward-in-time migration rates over time from the forward migration rates and the *Ne* trajectories using Eq 1, which expresses the backward-in-time migration rates as forward-in-time rates multiplied by the ratios of *Ne*’s in the source and sink location.

Next, we simulate one phylogenetic tree using 800 leaves, 400 from each location, and infer the *Ne* trajectories and migration rates using MASCOT-Skyline from that tree. We use an exponential distribution with a mean of 1 for the migration prior and the above specification of the GMRF for the *Ne* prior. We repeated this process 100 times.

**Fig 1:**
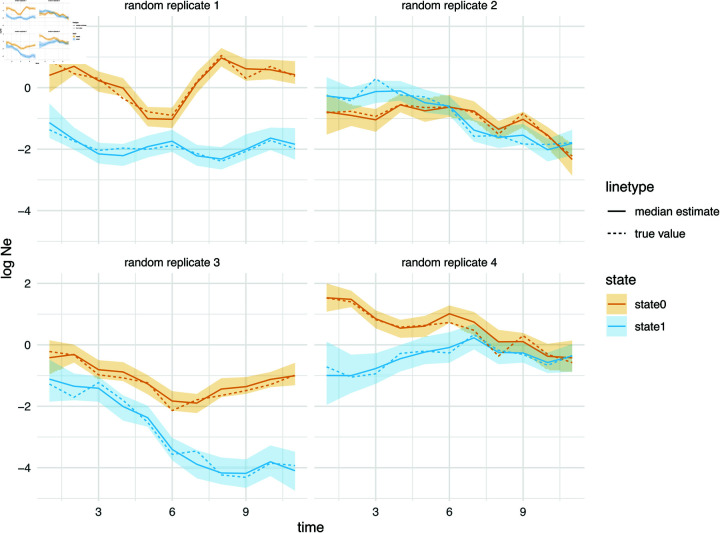
Inferred effective population size trajectories from simulated data. Here, we show the inferred effective population size dynamics with the line denoting the median inferred log *Ne*’s. The shaded areas denote the bounds of the 95% highest posterior density interval. The plots show the results for four of the 100 replicates chosen randomly.

In Fig 1, we show, for four of the total 100 randomly chosen replicates, that MASCOT-skyline can retrieve these nonparametric population dynamics from phylogenetic trees. Using these simulations, we obtain a 94% coverage of the 95% highest posterior density interval (HPD) of the true *Ne* value (see S1A Fig). The forward-in-time migration rates are also recovered well by MASCOT-Skyline (see S1B Fig), though, at 89%, the coverage is below the expected range (91% to 99%) of coverage estimates for 100 replicates. This is not unexpected as MASCOT is an approximation of the structured coalescent [45]. As shown in S2 Fig, MASCOT-Skyline is also able to recover population dynamics nested within the non-parametric effective population sizes, such as constant population sizes.

### Assumptions about the population dynamics drive ancestral state reconstruction in structured coalescent models

Spatial and temporal population dynamics impact the shape of phylogenetic trees. As such, we can expect methods that infer one dynamic aspect while ignoring the other may be biased.

To explore the nature of the bias, we use two simple examples. We simulated a phylogenetic tree using the exponential coalescent without any population structure (see S3 Fig) and one with two states that are both equally exponentially growing (see S4 Fig). Subsequently, we inferred the effective population sizes, migration rates, and internal node states twice, first assuming constant effective population sizes over time, and then allowing them to grow exponentially. In both cases, we model two demes, though in the first scenario, one is an unsampled ghost deme [47,48]. When not accounting for population dynamics, internal nodes deeper in the tree are inferred to be in a location other than the location of the samples (see S3A Fig). The effective population size of that second location is inferred to be much smaller than the first location (see S3B Fig). The smaller effective population size roughly corresponds to the effective population size early during the exponential growth (S3E Fig). The backward-in-time migration rates are inferred to be much higher from the sampled into the unsampled location than vice versa (S3C Fig). This pattern can also be observed when we have samples from both demes but do not account for population dynamics. Without correctly accounting for population dynamics the unstructured exponentially growing population is explained by a small population with strong migration into a larger population (see S4A&S4B Fig). Based on this illustration, we would expect to overestimate the number of introductions from a deme with few into a deme with many samples. When a deme has only a few samples, the effective population size of that deme essentially becomes unconstrained by any data, which the model will use to approximate past population dynamics.

We illustrate this issue using ZIKA virus (ZIKV) data and show how accounting for population dynamics can recover more plausible ancestral state reconstructions. We use a previously analyzed dataset of ZIKV sequences sampled from Polynesia, Brazil, the Caribbean, and various locations in South America [49]. This study used DTA to infer that ZIKV was most likely introduced once into the northeast of Brazil, followed by subsequent spread in Brazil and elsewhere in the Americas [49–51]. We perform two different inferences: first, we assume the effective population sizes to be constant over time, and second, we allow them to vary over time. We jointly infer the phylogenetic tree, evolutionary rate, and population parameters under the structured coalescent, assuming constant forward-in-time migration rates.

**Fig 2:**
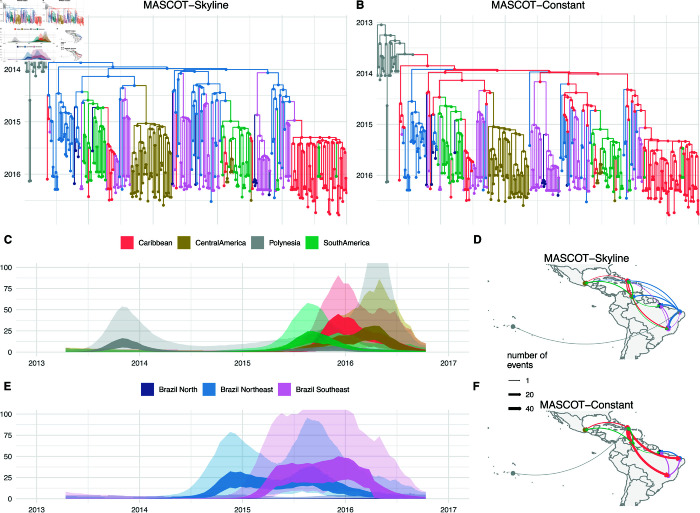
Inferred transmission dynamics of ZIKV when having skyline or constant *Ne* dynamics. **A** Inferred node states when inferring non-parametric skyline *Ne*’s in different demes. The tree is the maximum clade credibility (MCC) tree, and the nodes are colored by the node with the highest posterior probability in the MCC tree. **B** Inferred node states when each location has a constant *Ne* over time. **C & E** Inferred *Ne* trajectories for MASCOT-Skyline. The inner interval (dark) denotes the 50% highest posterior density (HPD) interval, and the outer (light) the 95% HPD interval. Inferred number of migration events between the different locations using MASCOT-Skyline **D** and MASCOT-Constant **F**. The maps are plotted using the R package maps [46], which sources the vector data from the ’Natural Earth data project’.

As shown in Fig 2A and 2B, the ancestral state reconstructions vary greatly when accounting for population dynamics (Skyline) and when not (Constant). Both methods put the root into Polynesia with over 90% probability. In the skyline scenario, we then infer one introduction from Polynesia to the northeast of Brazil and from there to the other parts of Brazil and the Americas. In the constant scenario, on the other hand, we infer an introduction of ZIKV from Polynesia to the Caribbean and subsequently to different regions in Brazil (see Fig 2B and 2F).

### Population structure biases population dynamic inference

As previously shown [52], population structure can impact the inference of population dynamics in coalescent skyline approaches. In particular, reductions in the effective population sizes towards the present can signal sub-population structure that is not accounted for [52].

We first show what effective population size dynamics a skyline method estimates when we do not model population structure, using the same simulations as in Fig 1. As we show in S5 Fig, ignoring population structure in these simulations means that the inferred effective population size trajectories closely resemble the larger population. We subsequently tested the scenario where we have simulations under a two-state model, but only sample from one deme. We randomly sampled and then re-inferred the *Ne* trajectories for the sampled and ghost demes, as well as the migration rates between them. As shown in S6 & S7 Figs, MASCOT-Skyline is able to retrieve both the *Ne* trajectories in both demes and also the migration rates from the ghost demes into the sampled deme. Migration rate estimates from the sampled deme into the ghost deme are inferred with wide HPD intervals, reflecting an absence of observed events from the sampled deme into the ghost deme (see S7 Fig).

To illustrate these patterns further, we compare how the inference of population dynamics is impacted when outside introduction into that population is not accounted for. To do so, we compiled a dataset with influenza A/H3N2 sequences sampled only from New Zealand and Australia, which we denote below as Oceania, sampled between 2000 and 2005. Oceania is thought to mainly act as a sink population for influenza A/H3N2, where there are introductions of viruses into the country that spark annual influenza epidemics, but viruses circulating in Oceania rarely seed epidemics elsewhere in the world [53,54].

Using this example dataset, we inferred the population dynamics in Oceania twice. First, we assumed no introduction of viruses into Oceania and no export of viruses out of Oceania. We then inferred the effective population size of influenza A/H3N2 in Oceania over time. Next, we allowed for an outside deme to represent influenza transmission anywhere outside of Oceania. This outside deme, sometimes referred to as a ghost deme [47,48], does not have any sampled sequences in the dataset. We estimated the effective population sizes of the outside deme over time alongside the migration rates between Oceania and the outside deme.

**Fig 3:**
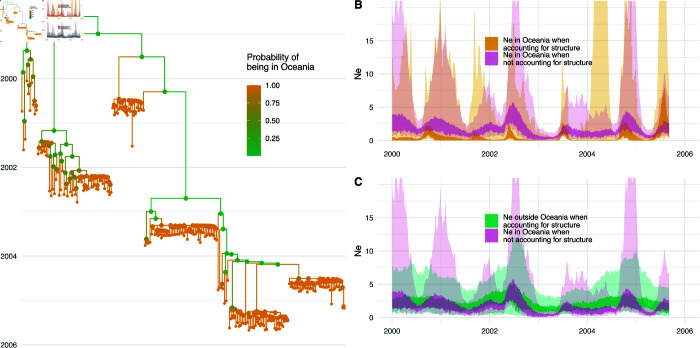
Misinterpretation of population structure as population dynamics for H3N2 in Oceania. **A** Inferred phylogenetic tree of 200 influenza A/H3N2 sequences sampled in New Zealand and Australia (Oceania). The probability of being in Oceania is computed as the average probability of a clade in the maximum clade credibility tree to be in Oceania. **B** Inferred effective population sizes in Oceania when allowing for an unsampled outside (ghost) deme and when assuming no population structure. **C** Inferred effective population sizes in Oceania when not allowing for an unsampled outside (ghost) deme compared to the inferred effective population size of the ghost deme when allowing for population structure.

As shown in Fig 3, the effective population size estimates are substantially different if we allow for an outside (ghost) deme compared to when we do not allow for that deme. If we allow a ghost deme, the inferred seasonality is much more pronounced. On the other hand, if we do not allow for a ghost deme, we see that the inferred effective population size dynamics of Oceania resemble the dynamics of the ghost deme more than the ones in Oceania.

### Sampling bias impacts ancestral state reconstructions

The coalescent patterns in phylogenetic trees indicate where lineages are over time. For example, rapid coalescence indicates smaller populations. If lineages rapidly coalesce, they are more likely to be in a smaller population. Further, what we typically consider a discrete location may have unaccounted-for sub-populations, potentially impacting analyses.

To investigate these patterns, we performed a simulation study to emulate a scenario where we have a pathogen transmitted in a large population with occasionally oversampled regional outbreaks in the same population, combined with spillovers with limited outbreaks into a second population. To this end, we simulated outbreaks using 40-state SIR models. The first state represents a large background deme, while the other demes experience small outbreaks. States 2 to 20 model local outbreaks in the same ’host’ species as the background deme, and states 21 to 40 represent outbreaks similar to the human outbreaks in the MERS-CoV scenario described below. We assume that cross-deme transmission events only occur from the background demes to the localized outbreak demes. We then reconstructed the transmission dynamics using MASCOT-Skyline between the compartments mimicking transmission in camels and those representing transmission in humans for different sampling rates in camels and humans. As shown in S8 Fig, MASCOT-Skyline correctly reconstructs transmission to occur almost exclusively from the source to the sink population. However, when there are only a few coalescent events in the tree representing transmission in the sink population, that pattern switches (see S8 Fig). This pattern is most pronounced if there are fewer than 10 coalescent events in the sink population. DTA only infers no events from the sink into the source when there are almost no samples from the sink population (see S9 Fig).

To illustrate these patterns in a real case scenario, we investigate the transmission of MERS-CoV between camels and humans using the dataset from [26]. MERS predominantly circulates in camels with occasional spillovers followed by limited transmission in humans. The dataset described in [26] contains 274 sequences sampled from humans and camels. The full dataset contains 174 sequences isolated from humans and 100 isolated from camels. We subsampled this dataset, ranging from 100% of human samples and 10% of the camel samples to 100% of both and then to 10% of the human samples and 100% of the camel samples. We then performed ancestral sequence reconstruction using MASCOT-Skyline and DTA with a skygrid coalescent prior [19], using a strict clock with an HKY+$\Gamma_{4}$ site model.

**Fig 4:**
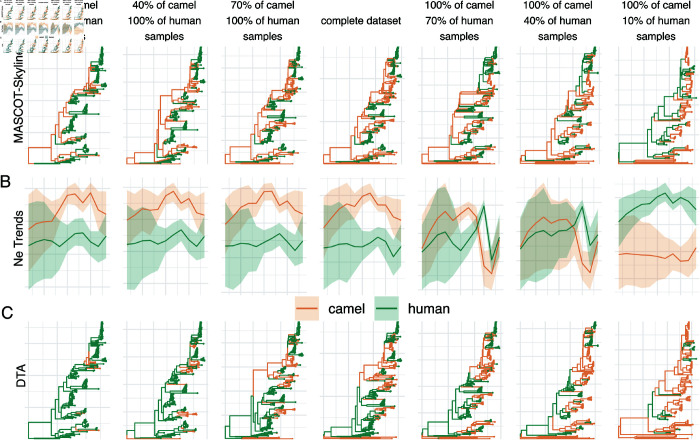
Repeated spillover of MERS-CoV from camels to humans. **A** Maximum clade credibility (MCC) trees inferred using MASCOT-Skyline for different amounts of samples from camels and humans, from left to right. Each branch is colored by the most likely location of the child node of that branch. **B** Inferred effective population size trajectories using MASCOT-Skyline for different amounts of samples from camels and humans. **C** Maximum clade credibility (MCC) trees inferred using DTA.

As shown in Fig 4, when there are few camel samples, DTA infers MERS to circulate in humans with occasional spillovers into camels. With all 274 sequences in the data, DTA still infers the predominant circulation in humans. DTA starts to infer that MERS-CoV circulates predominantly in camels when most human samples are removed from the analysis. Both methods infer similar tree structures as measured by the tree height and length (see S10 Fig)

Conversely, MASCOT-Skyline infers predominant circulation in camels, even if most camel sequences are removed. The reason for that is that the human samples indicate rapid coalescence and, therefore, a small *Ne*. For branches that do not conform with a small *Ne*, it infers them to be in the larger outside (here camel) population. When we remove more and more human sequences, the picture changes. The more recent camel sequences are strongly clustered geographically, also indicating a small *Ne*. Now that there are fewer human sequences, the human *Ne* effectively takes the role of a “ghost” deme, and MASCOT-Skyline infers rapid coalescence (that is, the local outbreak clusters) after introductions from elsewhere. Since the only possible location for elsewhere is the human compartment, MASCOT-Skyline infers that local outbreak clusters have been introduced from outside. Interestingly, this means that the biases are inverted between the MASCOT-Skyline and DTA, with MASCOT-Skyline being more likely to infer a human source with fewer human samples.

We next remove local outbreak clusters by first identifying groups of sequences sampled from the same location in the same month. We then only use one of the sequences from that group to represent the outbreak. When we remove local outbreak clusters in the camel compartment, we infer camels to be the source location much more consistently across different sample numbers (see S11 Fig). We infer circulation in humans only when using almost exclusively camel sequences.

### Modeling population size dynamics is necessary to reconstruct migration rates

When we analyze spatial transmission patterns, we typically seek to infer the movement of viral lineages and/or the rates governing that movement. Reconstructing the movement of viral lineages—performing ancestral state reconstruction—can reveal how many introductions occurred in a location and the number of migration events between locations. However, the number of events identified directly correlates to the number of samples in a location. The more we sample from a location, the more introductions into that location we will identify. The migration rates are population-level parameters independent of the number of samples. The migration rates also tend to be more important to understanding the spread of pathogens than solely the number of migration events.

Importantly, migration rates can be used to determine what drives spatial transmission dynamics, such as using generalized linear models (GLM) [55]. In the GLM approaches [55,56], the contribution of predictors to the migration rates is inferred instead of directly inferring these rates. Yet, this still relies on the models’ ability to quantify migration rates accurately.

To explore the behavior and biases of DTA and MASCOT-Skyline to recover migration rates, we perform simulations using a Susceptible-Infected-Recovered (SIR) model with two states using MASTER [44]. We perform SIR simulations using various sampling models, different migration rates, and different reproduction numbers R0 across states.

MASCOT-Skyline is able to recover the prevalences over time for the two states (see S12 & S13 Figs). Both methods, DTA and MASCOT-Skyline, can recover ancestral states similarly well for low rates of migration (see S14 Fig). DTA has greater posterior support for both the right and the wrong node states (see S14 Fig). Overall, both approaches recover the true ancestral node states similarly well.

However, we find large differences in the migration rate estimates between the two methods (see S15 Fig). MASCOT-Skyline recovers the rates accurately for most simulation scenarios, with somewhat worse performance when R0s differ across the two states (see S15 Fig). This was expected based on our assumption that the prevalence is proportional to the effective population size with the same proportionality factor across states. We therefore expect that explicitly accounting for differences in these proportionality factors would remedy these biases. DTA overall suffers from relatively low coverage of the true value in these simulations, between 27% and 89%. These low coverage values are partly explained by a lower correlation between true and estimated values, but also by narrower highest posterior density intervals (see S16 Fig). Both methods are able to retrieve the magnitude of migration, that is, the mean migration rate accurately (see S17 Fig). The estimated mean migration rates are highly correlated with the simulated values, though DTA has lower coverage of the true simulated values due to narrower HPD intervals. Next, we compared the ratio of migration rates from state 1 to 2 over the migration rate from 2 to 1. Lastly, we investigate whether correcting for the cumulative prevalence in the source and sink locations for DTA improves the correlation of the migration rate estimates. We find some improvement, but the correlation is still weaker than for MASCOT-Skyline (see S18 Fig).

We next show, using the example of SARS-CoV-2, how these patterns can impact the interpretation of migration patterns. We use sequences collected from Washington state (USA), North America, and the rest of the world, previously analyzed in [57]. We further split sequences in Washington state into eastern and western Washington state based on whether the county of isolation is east or west of the Cascade mountain range. The idea is to have different demes, where we know the population sizes and can have expectations about reasonable migration rates. We then performed phylogeographic analyses using MASCOT-Skyline and DTA using a skygrid coalescent prior. For both analyses, we used a strict clock model and an HKY+$\Gamma_{4}$ site model.

**Fig 5:**
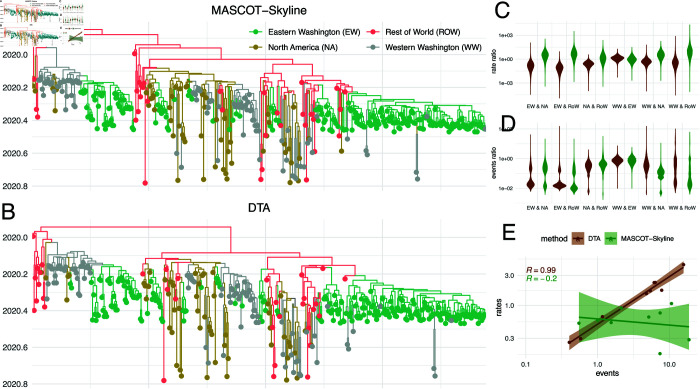
Reconstruction of the geographic spread of SARS-CoV-2 between the world, North America, and Eastern and Western Washington. **A** Maximum clade credibility tree reconstructed using MASCOT-Skyline and DTA (**B**). The colors represent the inferred node states with the highest posterior probability. **C** Inferred migration rate ratios between the four locations using MASCOT-Skyline and DTA. Each violin plot shows the rate ratio from A to B over the rate of B to A. **D** Inferred number of migration events between the four locations using MASCOT-Skyline and DTA. Each violin plot shows the number of migration events from A to B over the number of events from B to A. **E** Correlation between the inferred migration rates and the number of migration events between the four locations. The correlation coefficients are calculated using the median number of events between the 4 locations and the median migration rates between them.

As shown in Fig 5A and 5B, DTA and MASCOT-Skyline infer similar ancestral state reconstructions. These similar ancestral state reconstructions reflect similar migration events between the four discrete locations (Fig 5D). To further quantify the similarity in the ancestral state reconstructions between the two methods, we infer the sampling location of 5 random tips from each location that have had their location masked before running phylogeographic inference. We then computed the posterior support of the sampled location to be in the correct location of isolation. As shown in S19 Fig, the posterior support for the correct location of isolation is similar between the two methods. However, DTA has a higher posterior support for the actual sampled location than MASCOT-Skyline.

While the two methods reconstruct similar ancestral states, they infer vastly different migration rates (Fig 5C). In particular, DTA infers migration rates highly correlated to the number of migration events between two locations (Fig 5E). The migration rates inferred by MASCOT-Skyline instead have little to no correlation with the number of migration events between the two locations. If we have two locations, one with ten times more infected individuals, then we would expect ten times more migration events from that location, even if the migration rates are the same. Therefore, this means that the number of migration events is not a sufficient measure of the migration rates. Since DTA does not incorporate population dynamics into the estimation of migration rates, these differences are not unexpected.

## Discussion

Here, we show that population dynamics and population structure are intrinsically linked when inferring the spread of pathogens. This is consistent with previous work on biases in phylogeographic [39] and phylodynamic models [52]. To address this, we develop MASCOT-Skyline, an approach to infer non-parametric population dynamics alongside population structure.

Using the example of ZIKV spread in South America, we show that assuming the wrong population dynamic model dramatically impacts the reconstruction of how the spread of ZIKV unfolded, with MASCOT-Skyline providing a reconstruction that is much more consistent with other estimates [49].

The bias introduced by assuming constant effective population sizes over time is relatively hard to predict *a priori*. Anecdotally, locations with very few samples can act similarly to a ghost deme [47,48]. In that case, the *Ne* of locations with only a few samples is potentially used by the model to approximate the population dynamics of the other state. While beyond the scope of this paper, the impact of modeling population size dynamics in a parametric versus non-parametric way remains an open question. Methods exist to, for example, compute effective population size trajectories from deterministic SIR trajectories [37]. In particular, it will be interesting to see at what point assuming deterministic dynamics to approximate stochastic dynamics breaks ancestral state reconstruction.

While we did not explicitly investigate performance differences between MASCOT-Skyline and constant, the computational demands for MASCOT-Skyline do not seem to be substantially higher than for the constant approach. The ZIKV example, which ran for about 1 week, had approximately half the posterior ESS (952 vs. 541) for MASCOT-Skyline than for MASCOT-Constant on 250 samples and 7 states with 26 *Ne*’s being estimated per state. When the *Ne* is only estimated at, for example, ten or fewer time points, we expect this difference to narrow. Population dynamics are present to some degree in most datasets, which should make approaches that account for them better suited to analyze these datasets in all but a few cases. We confirm the similar computational demands of the MASCOT-Skyline and Constant using simulations for datasets with population dynamics (see S20 & S21 Figs, which also show the relationship between ESS per hour and number of samples and states). As such, we recommend defaulting to MASCOT-Skyline over constant.

Using the example of MERS-CoV, we illustrate this bias by changing the number of samples from humans and camels. MERS-CoV circulates in camels and repeatedly spills over into humans, causing limited outbreaks. If the sink population is extremely undersampled, the effective population size of the sink population will be used to approximate the population dynamics of the source population. Interestingly, this leads to the opposite sampling bias than in the case of discrete trait analyses (DTA) [29]. DTA tends to assign the source location to the overrepresented deme. The fewer human samples there are, the more likely camels are inferred to be the source location. This is caused by the sampled numbers being treated as informative by DTA. While the explanation for the pattern inferred by DTA is relatively straightforward, the explanation for the pattern inferred by MASCOT-Skyline is more complex. We suspect that with more camel samples and only a few human samples, MASCOT-Skyline infers a *Ne* trajectory for the camel state that is consistent with the local outbreak clusters. This opens interesting questions about what level of structure is important to consider in such analyses and how to choose samples that reflect that level of population structure. In the case of MERS-CoV, if one is interested in the structure at the level of the host species, sampling (or subsampling) has to be performed to represent this structure. As such, doing so requires information about the sampling process and the potential exclusion of some of the sequences collected, for example, from outbreak clusters. A limitation of our MERS analysis is that both methods assume state changes along branches instead of at transmission events, which means that state changes at internal nodes are not allowed, presenting a model misspecification.

Ancestral state reconstruction provides a picture of the path of individual lineages. Additionally, ancestral state reconstructions can act as a sanity check on whether the inference results are consistent with prior knowledge, such as which species is the host species. Using the example of SARS-CoV-2, we show that similar ancestral state reconstructions can lead to vastly different migration rate estimates between DTA and MASCOT-Skyline. While we do not know the true migration rates in this case, the rate estimates of MASCOT-Skyline are more consistent with what is expected from the population sizes of the different locations in the dataset.

Based on our simulation study and the SARS-CoV-2 example, the migration rate estimates by DTA should likely not be interpreted as population-level parameters in most cases. That is, they do not reflect the rate at which an individual in location A moves to location B unless the sampling rates are constant over time and the same across locations A and B. Instead, they should likely be interpreted as a parameter that mainly reflects the observed number of migration events between locations A and B in the dataset. Therefore, subsampling strategies for DTA analyses should likely be based on the number of infected over time and across locations. If that is not possible, the migration rate estimates may not be directly interpretable as epidemiological parameters. This also poses interesting questions for methods that seek to reconstruct the drivers of migration patterns, for example, using generalized linear models [55,56]. The results of such analyses could also be subject to similar limitations.

Overall, our work shows that to reconstruct the rates at which pathogens move across demes, such as geographic locations or host types, one has to consider the population dynamics in those demes.

## Methods and materials

### MASCOT

MASCOT, the marginal approximation of the structured coalescent, tracks the probability of lineages being in any of the modeled states backward in time by solving ordinary differential equations described in [45] and [40]. MASCOT is parameterized by effective population sizes and migration rates. The effective population size of state *a* is given by *Ne*_*a*_, and the backward migration rate from state *a* to state *b* is given by *m*_*ab*_. MASCOT assumes that the effective population sizes and migration rates are constant during each integration step. By solving the ordinary differential equations (ODEs), MASCOT computes the probability of the tree given the parameters $P(T|\vec{Ne},\vec{m})$. To model time-varying parameters, we feed the continuously varying values for $\vec{Ne(t)}$ and $\vec{m(t)}$ into the ODE calculations as piecewise constant values $\vec{Ne(t)}$ and $\vec{m(t)}$ at different time points *t* that approximate the underlying continuous dynamics. The piecewise constant approximation uses a user-defined number of intervals, with more intervals leading to a better approximation of the continuous dynamics of the parameters, but also higher computational costs. We further explain this in S22 Fig. The probability $P(T|\vec{Ne},\vec{m})$ can then be computed by integrating over all possible states at the root of the tree [40]. Additionally, one can compute the probability of each node in the tree being in any state to perform ancestral state reconstruction [40] or explicitly reconstruct the migration histories using stochastic mapping [58].

### MASCOT-Skyline

To model nonparametric population dynamics alongside population structure, we first define a grid of time points to model nonparametric population dynamics. We define the grid in absolute time or relative to a tree’s height, which is the default option. We then infer each grid point’s effective population size *Ne*_*a*_(*t*). Between those points, we assume that the effective population sizes change continuously according to an exponential growth model. Effectively, we use linear interpolation between any two adjacent *Ne*’s in log space. Alternative approaches, such as spline interpolation, would also be possible to implement. For the computation of $P(T|\vec{Ne(t)},\vec{m(t)})$, we approximate the continuous parameter dynamics using piecewise constant approximation as described above and then use the piecewise constant values for the integration of the MASCOT ODEs. Typically, the number of intervals used for the piecewise constant approximation should be substantially higher than the number of *Ne*’s estimated for this to be a reasonable approximation.

By default, we assume the forward-in-time migration rates to be constant over time. As the backward-in-time migration rates that go into the computation of $P(T|\vec{Ne(t)},\vec{m(t)})$, we say that the backward-in-time (bw) migration rate $m_{ab}^{bw}$ from *a* to *b* can be expressed from the forward-in-time (fw) migration rate $m_{ba}^{fw}$ as:

$$m_{ab}^{bw}(t)=m_{ba}^{fw}\frac{Ne_{b}(t)}{Ne_{a}(t)}$$ (1)

Using the derivation of the coalescent rates or effective population sizes in [12], the error ϵ of this assumption is:


$$m_{ba}^{fw}\frac{Ne_{b}(t)}{Ne_{a}(t)}=\epsilon m_{ba}^{fw}\frac{\frac{I_{b}(t)}{\beta_{b}\frac{S_{b}}{N_{b}}}}{\frac{I_{a}(t)}{\beta_{a}\frac{S_{a}}{N_{a}}}}$$

with *I*_*a*_(*t*) being the number of infected individuals, *S*_*a*_(*t*) the number of susceptible individuals, and *N*_*a*_(*t*) the total population size and $\beta_{a}$ the transmission rate of location *a* at time *t*. Therefore, we can compute the error ϵ introduced by the assumptions in Eq 1 as:


$$\epsilon =\frac{\beta_{a}\frac{S_{a}}{N_{a}}}{\beta_{b}\frac{S_{b}}{N_{b}}}$$

meaning the error we introduce equals the effective transmission rate in the sink divided by the rate over the sink. With the reduction of the pool of susceptible individuals *S*_*a*_, the error ϵ induced by differences in the transmission rates *β* will likely become smaller. Therefore, the error of the assumption that the ratio of *Ne*’s between source and sink is equal to the ratio between the number of infected individuals is reduced over time. However, in cases where there is a difference in, for example, the generation time (or the becoming-uninfectious rate), the error will persist. For example, this could be the case when studying the transmission across different host species.

In addition to the skyline model, we implemented exponential and logistic growth models. The different dynamic models for the effective population size can be mixed. For example, state *a* can be a skyline model, while state *b* can grow exponentially or be constant. The above equation assumes that the ratio of Ne’s between source and sink locations is equal to the ratio of the number of infected individuals.

### Joint inference of effective population sizes and migration rates

To infer the effective population sizes, the different demes, and the migration rates, we use the adaptable variance multivariate normal operator [42]. The adaptable variance multivariate normal operator proposes new parameter states during the MCMC and learns the correlation structure between the different parameters. The effective population sizes are denoted in log space, while the migration rates are estimated in real space. The prior on the effective population sizes, sometimes referred to as a smoothing prior, is similar to the skyline method [19]. The implementation of the smoothing prior works is as follows. One can choose an arbitrary prior on the difference between two adjacent *Ne*’s in log space. If we want the smoothing prior to being a Gaussian Markov random field (GMRF), we can assume that the *Ne* at time *t* + 1 *Ne*(*t* + 1) = 𝒩(Ne(t),σ), that is normally distributed around *Ne*(*t*) with standard deviation *σ*. *σ* can be fixed or estimated from the data, which corresponds to the precision of the skyline method [19]. By default, selecting the *σ* to be estimated for each state will mean that a different value for *σ* will be estimated individually for each state. To change between source and sink locations throughout the MCMC, we use an operator that swaps the effective population sizes for the same time points between locations *a* and *b*. All other operators used for the MCMC are the default operators in BEAST2 [43].

### Implementation

We implemented MASCOT-Skyline as part of the BEAST2 package MASCOT. MASCOT-Skyline requires at least the BEAST2 version 2.7 to execute. The code is available at https://github.com/nicfel/Mascot and through the BEAST2 package manager. MASCOT-Skyline is implemented in Java. MASCOT-Skyline is available starting from MASCOT version v3.0.5. Analyses can be set up using the BEAUti interface of BEAST2 by choosing MASCOT-Skyline as a tree prior. The effective population size dynamics are chosen separately for each location, deme, or state. Therefore, constant, exponential, or skyline effective population size dynamics can all be used in the same analyses, albeit for different states. For setting the specifications of the Gaussian Markov Random Field (GMRF) prior on the skyline dynamics, one has to specify the prior on the difference between adjacent *Ne*’s (that is, between the *Ne* at time *t* and at time t+1) to a normal distribution with mean 0 and standard deviation *s*. The standard deviation can then be specified or estimated. The standard deviation is estimated, by default, individually for each state. Throughout this paper, we assume that each state’s standard deviation is the same. For the exponential growth model (tested in S23 Fig), we *a priori* assume that the log *Ne* at the time of the most recent sample and the growth rates are normally distributed. Implementing MASCOT-Skyline as an open-source package to BEAST2 allows users to use a variety of evolutionary models and data sources implemented in BEAST2 or packages to BEAST2, including relaxed clock models or amino acid alignments.

### SIR simulation study

We use a two-state model to aid the interpretability of the results. We simulate outbreaks in two states, each with an R0 of 1.5, a recovery rate of 52, and a random total population size sampled from a uniform distribution between 500 and 10000. The migration rates are sampled from an exponential distribution with a mean of 5 (low migration rate scenario) or 25 (high migration rate scenario). We simulate phylogenetic trees using the SIR model in MASTER [44]. We then use either 250 or 500 samples per state for inference from the phylogenetic trees. Or use a constant sampling rate, conditioning on at least 50 samples per state. On average, the simulations had 389 (low migration) and 431 (high migration) tips. In the constant sampling scenario, we simulated trees with 4000 tips per state. We then subsampled the tips to have 250 samples per state, sampled evenly across time. Importantly, the samples per state will potentially impose implicit constraints on the possible values for other simulation parameters, such as a state’s population size.

We performed discrete trait analyses (DTA) using the BEAST v1.10.4 [59,60]. For all analyses, we use a coalescent skygrid tree prior [19]. We estimate the mean migration rate and the relative migration rates between locations. We use an exponential prior on the mean migration rate. We use either 5 (low migration rate scenario) or 25 (high migration rate scenario) for the mean of the exponential prior. We use an exponential prior with a mean of 1 for the relative migration rates. This parameterization of the migration rates is necessitated by the parameterization of the DTA likelihood calculation, which normalizes relative migration rates.

Next, we infer the migration rates and the effective population size dynamics using MASCOT-Skyline. We use a Gaussian Markov Random Field (GMRF) smoothing prior to the *Ne*’s over time and estimate the variance. We estimate the *Ne* at 26 points in time. For the migration rates, we use an exponential distribution with the mean equal to the mean migration rates in the simulations, i.e., 5 or 25.

### Software

All other plots are done in R using ggplot2 [61], ggtree [62], and ggpubr [63]. Convergence is assessed using conda [64].

## Supporting information

S1 Fig Inferred vs. true effective population sizes and forward in time migration rates for non-parametric *Ne* dynamics. **A** Inferred vs. true effective population size estimates. **B** Inferred vs. true forward in time migration rates. The coverage (cov) of the true value by the 95% highest posterior density interval is shown on the top. The coverages are computed from the 100 simulations, two states, and the 11 separately estimated, but correlated *Ne*’s per state and simulation. The root mean squared errors (RMSE) are computed from the difference between the median log *Ne* and the true log *Ne*, and the median log migration rates and true log migration rates.
(PDF)

S2 Fig Inferred non-parametric effective population sizes for populations simulated using constant effective population sizes. Here, we show how MASCOT-Skyline recovers the effective population sizes when the underlying dynamics are constant.
(PDF)

S3 Fig Misinterpretation of population dynamics as population structure when allowing for a ghost deme. **A** Inferred node states when assuming a two-state structured coalescent model with two constant populations. **B** Inferred effective population sizes of the two populations. The dotted black line denotes the true *Ne* of the orange population. **C** Inferred migration rates between the two constant populations. **D** Inferred node states when assuming a two-state structured coalescent model, allowing the two states to grow exponentially. **E** Inferred effective population sizes over time of the location where all samples were taken from (orange). The *Ne* of the blue location is sampled under the prior and, therefore, not shown in the figure. **F** Migration rates between the location where samples were taken and a second (blue) location.
(PDF)

S4 Fig Misinterpretation of population dynamics as population structure when all demes are sampled. **A** Inferred node states when assuming a two-state structured coalescent model with two constant populations. **B** Inferred effective population sizes of the two populations. The dotted line denotes the true *Ne* of the blue and the orange population. **C** Inferred migration rates between the two constant populations. **D** Inferred node states when assuming a two-state structured coalescent model, allowing the two states to grow exponentially. **E** Inferred effective population sizes over time of the location where all samples were taken from (orange). **F** Migration rates between the location where samples were taken and a second (blue) location.
(PDF)

S5 Fig Inferred *Ne* trajectories for a two-state structured coalescent model when population structure is ignored. Here, we infer the effective population size (*Ne*) trajectory for tree simulation under a two-state structured coalescent model with time-varying population size. We do so once modeling the two states (state 0 in orange and state 1 in blue) and once ignoring any population structure (combined in green).
(PDF)

S6 Fig Inferred vs. true effective population sizes and forward in time migration rates for non-parametric *Ne* dynamics for two state simulations where one deme is unsampled. **A** Inferred vs. true effective population size estimates in the sampled deme. **B** Inferred vs. true forward in time migration rates from the ghost (unsampled) deme into the sampled deme. **C** Inferred vs. true effective population size estimates in the ghost deme. **B** Inferred vs. true forward in time migration rates from sampled deme into the ghost deme. The coverage (cov) of the true value by the 95% highest posterior density interval is shown on the top.
(PDF)

S7 Fig Inferred *Ne* trajectories for a two-state structured coalescent model when population structure is ignored, but we only sample from one deme. Here, we infer the effective population size (*Ne*) trajectory for tree simulation under a two-state structured coalescent model with time-varying population size. Only one of those demes (orange) is sampled, while the other one is unsampled (ghost deme). We do so once modeling the two demes (sampled deme in orange and ghost deme in blue) and once ignoring any population structure (in green). The coverages for the *Ne*’s are computed for all 11 separately estimated, but correlated *Ne*’s for the 100 simulations.
(PDF)

S8 Fig Inferred source-sink dynamics relative to the true number of coalescent events observed in the sink population. Here, we simulate transmission histories under a 40-state SIR model. The first state is considered the source species and has a population size of 10000 and an R0 of 1.5. States 2 to 20 model localized and oversampled outbreaks in the source species, and each state has a population size of 20 and an R0 of 5. Each of the 20 sink populations has a population size of 5 and an R0 of 10 to model rapid, but contained outbreaks. Transmission, or spillover, can only happen from state 0 to any other state, but not backward. We then vary the amount of sampling in the source species and the sink species. On the x-axis, we show the number of true coalescent events captured in the simulated transmission histories that happened in any of the sink states. On the y-axis, we show the number of inferred migration events from the source to the sink and from the sink to the source. The latter is, in the simulations, always 0, but is inferred to be highly above 0 when there are only a few coalescent events in the sink, and the sink essentially starts to act as a ghost population.
(PDF)

S9 Fig Inferred source-sink dynamics using DTA depending on the number of samples in the sink population. Using the same situations as for S8 Fig, we reconstructed the source sink transmission dynamics using DTA. The y-axis shows the number of inferred transmission events. The true number of transmission events from the sink to the source is 0. The x-axis shows the number of samples in the sink population.
(PDF)

S10 Fig Comparison of the tree height and length distributions between MASCOT-Skyline and DTA. Here, we compare the tree heights and lengths between DTA and MASCOT-Skyline for the different subsampled datasets of the MERS-CoV analysis. The dots denote the median estimate and the error bars the 95% HPD interval.
(PDF)

S11 Fig Repeated spillover of MERS-CoV from camels to humans when removing local outbreak clusters. Here, we show the inferences of the transmission dynamics of MERS-CoV between humans and camels when we remove local outbreak clusters in the camel compartment, defined as sequences sampled from the same location in the same month. **A** Maximum clade credibility (MCC) trees inferred using MASCOT-Skyline for different amounts of samples from camels and humans, from left to right. Each branch is colored by the most likely location of the child node of that branch. **B** Inferred effective population size trajectories using MASCOT-Skyline for different amounts of samples from camels and humans. **C** Maximum clade credibility (MCC) trees inferred using DTA.
(PDF)

S12 Fig Simulated Prevalence for the two states in the SIR model, part 1. Comparison between simulated prevalences for the two states and the inferred log *Ne*’s for the two states using MASCOT-Skyline. The trajectories are shown for the first 5 runs of the simulation scenarios, denoted on the left.
(PDF)

S13 Fig Simulated Prevalence for the two states in the SIR model, part 2. Comparison between simulated prevalences for the two states and the inferred log *Ne*’s for the two states using MASCOT-Skyline. The trajectories are shown for the first 5 runs of the simulation scenarios, denoted on the left.
(PDF)

S14 Fig Distribution of the posterior support for the true node states inferred by DTA and MASCOT-Skyline. Here, we show the distribution of posterior support for the true node states for the different SIR simulation settings. The posterior node supports are shown DTA and MASCOT-Skyline. Each subplot uses different settings for the simulations: low or high migration rates, where the mean migration rate was 5, respectively. 25. 250 or 500 samples per state, or proportional.
(PDF)

S15 Fig Correlations between the simulated and inferred migration rates for MASCOT-Skyline and DTA. Here, we show the simulated (x-axis) and estimated (y-axis) migration rates using simulations under a two-state SIR model. The dots show the median estimate, and the error bars show the 95% highest posterior density (HPD) interval. The Pearson correlation coefficients (R) are calculated separately for MASCOT-Skyline and DTA. The coverage of the true value by the 95% HPD is shown after cov. The coefficients are calculated between the simulated values and the median estimates. Each subplot uses different settings for the simulations: low or high migration rates, where the mean migration rate was 5, respectively. 25. 250 or 500 samples per state, or proportional and constant sampling.
(PDF)

S16 Fig Relative HPD interval width for MASCOT-Skyline and DTA. Here, we show the simulated (x-axis) and estimated (y-axis) migration rates using simulations under a two-state SIR model. The dots show the difference between the upper and lower bounds of the 95% highest posterior density interval divided by the median estimate. The red horizontal line shows the line for the upper and lower bounds of the 95% interval of an exponential distribution used as a prior on the migration rates.
(PDF)

S17 Fig Estimation of the mean migration rate from two-state SIR simulations. Here, we show the simulated (x-axis) and estimated (y-axis) migration rates using simulations under a two-state SIR model. The dots show the median estimate, and the error bars show the 95% highest posterior density (HPD) interval. The Pearson correlation coefficients (R) are calculated independently for MASCOT-Skyline and DTA, are shown in the top left corner of each plot, and are computed between the log of the true value and the log of the median estimate. We additionally show how often the 95% HPD interval covers the true value (cov). The coefficients are calculated between the simulated values and the median estimates. Each subplot uses different settings for the simulations, i.e., low or high migration rates, where the mean migration rate was 5 resp. 25. 250 or 500 samples per state, or proportional and constant sampling.
(PDF)

S18 Fig Simulated and inferred migration rate ratios using two-state SIR simulations for MASCOT-Skyline and DTA. Here, we compare the simulated migration rate ratio to the estimated ratio of migration rates between the two states in the SIR model. The migration rate estimates are shown for DTA, MASCOT-Skyline, and DTA with case correction, where we multiply the ratio of migration rates by the ratio of cumulative incidence over the simulations to correct for differences in population size. The dots show the median estimate of the migration ratios, and the error bars show the 95% highest posterior density (HPD) interval. The Pearson correlation coefficients (R) are calculated independently for MASCOT-Skyline and DTA and DTA with case correction. The correlation coefficients are computed between the log of the true value and the log of the median estimate. We additionally show how often the 95% HPD interval covers the true value (cov). Each subplot uses different settings for the simulations, that is, low or high migration rates, where the mean migration rate was 5, respectively. 25. 250 or 500 samples per state, proportional, and constant sampling.
(PDF)

S19 Fig Posterior support for true tip state between MASCOT-Skyline and DTA. We compare the posterior support for the true sampling location inferred using MASCOT-Skyline and DTA for the four locations in our SARS-CoV-2 dataset. For the inference, the sampling location of random samples in the dataset was masked, and the location was re-inferred. The posterior support for the true location then denotes how much posterior weight the MCMC algorithm is putting on the inferred sampling location between the true sampling location. The dotted lines denote the percentage of samples from each geographic location that is in the analyses, i.e., a line at 0.25 would indicate that 25% of samples in the dataset are from that location.
(PDF)

S20 Fig Comparison of the computational speed of MASCOT-Skyline and MASCOT-Constant across a range of states and samples. Here, we compare the computational speed of MASCOT-Skyline and MASCOT-Constant for different-sized datasets. To compare the two methods, we simulated phylogenetic trees using a structured coalescent skyline model for different numbers of states and different numbers of samples. We then inferred the population dynamics from the true phylogenetic trees using MASCOT-Skyline and MASCOT-Constant. Next, we plot the ESS per hour of the posterior probability of MASCOT-Skyline over the ESS per hour of the MASCOT-Constant for inference from the same phylogenetic tree (y-axis). The x-axis shows the total number of samples or leaves in the simulation. The different colors show the number of demes in the simulations. **A** ESS_skyline_/ESS_constant_ per hour on a log-scale (y-axis) against the number of demographic states (x-axis). The color of the points indicates the total samples for each run. **B** ESS_skyline_/ESS_constant_ per hour (log-scale) against total samples. The color of the point indicates the number of states. **C** Absolute ESS per hour under the skyline model (log-scale) vs. number of states. **D** ESS per hour (skyline model, log-scale) vs. number of samples. **E** ESS per hour under the constant-population model (log-scale) vs. number of states. **F** ESS per hour (constant model, log-scale) vs. number of samples.
(PDF)

S21 Fig Comparison of the computational speed of MASCOT-Skyline and MASCOT-Constant for a range of samples and 2 states, but ultrametric trees. Here, we compare the computational speed of MASCOT-Skyline and MASCOT-Constant for different-sized datasets that are not sampled through time. As a result, the maximum number of co-existing sequences is the number of samples. We then inferred the population dynamics from the true phylogenetic trees using MASCOT-Skyline and MASCOT-Constant. Next, we plot the ESS per hour of the posterior probability of MASCOT-Skyline over the ESS per hour of the MASCOT-Constant for inference from the same phylogenetic tree (y-axis). The x-axis shows the total number of samples or leaves in the simulation. The different colors show the number of demes in the simulations. **D** ESS per hour (skyline model, log-scale) vs. number of samples. **F** ESS per hour (constant model, log-scale) vs. number of samples.
(PDF)

S22 Fig Description of how the effective population sizes are described over time. Each location in the dataset has its own population size trajectory. The population size trajectory is considered between the most recent sampled individual (mrsi) and the tree’s root. Here, we consider two more effective population sizes (*Ne*) between these two points in time. In this case, we estimate four *Ne*’s per location, with any number of *Ne*’s possible. Between the four points where we estimate the *Ne*, we assume that the *Ne* changes through exponential growth or decline. For the log of the *Ne*, that means we are using linear interpolation.
(PDF)

S23 Fig Parameter inference for three states with exponential growth. **A** Inferred vs. true effective log population size at the present. **B** Inferred vs. true growth rates. **C** Inferred vs. true forward in time migration rates. The coverage (cov) denotes how often the true, simulated value was part of the 95% highest posterior density intervals.
(PDF)

S1 Text SARS-CoV-2 GISAID acknowledgement table. (PDF)
