# Supplementary figures and images for: MASCOT-Skyline integrates population and migration dynamics to enhance phylogeographic reconstructions
Source: PLoS Comput Biol. 2025 Sep 26;21(9):e1013421. doi: 10.1371/journal.pcbi.1013421 (PMC12500135; doi:10.1371/journal.pcbi.1013421)

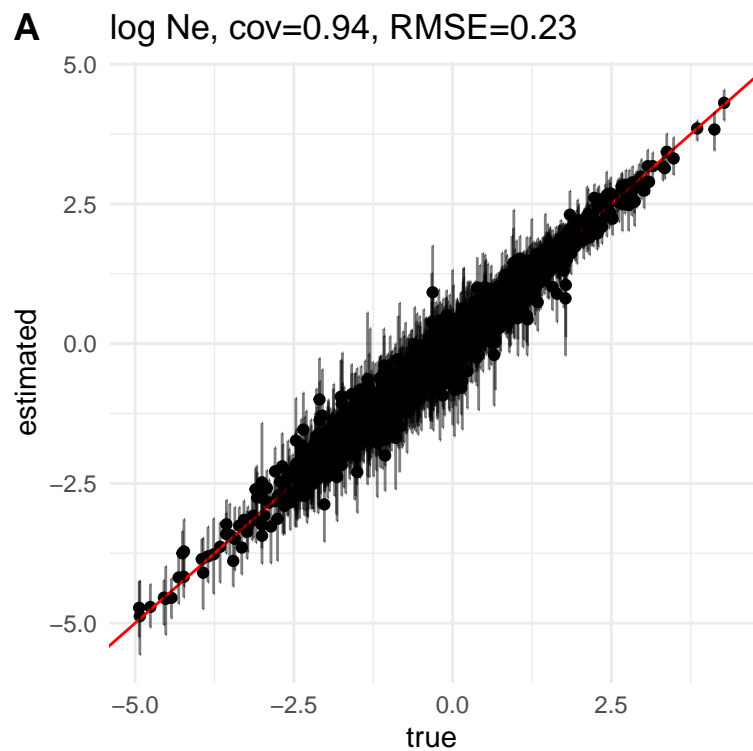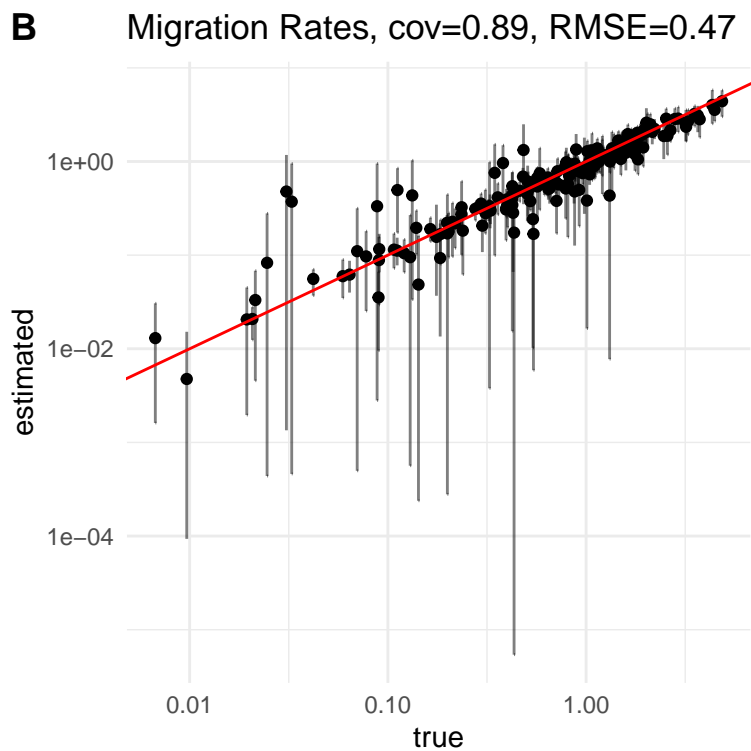

Supplement: S1 Fig — A Inferred vs. true effective population size estimates. B Inferred vs. true forward in time migration rates. The coverage (cov) of the true value by the 95% highest posterior density interval is shown on the top. The coverages are computed from the 100 simulations, two states, and the 11 separately estimated, but correlated Ne’s per state and simulation. The root mean squared errors (RMSE) are computed from the difference between the median log Ne and the true log Ne, and the median log migration rates and true log migration rates. (PDF) [file pcbi.1013421.s001.pdf]

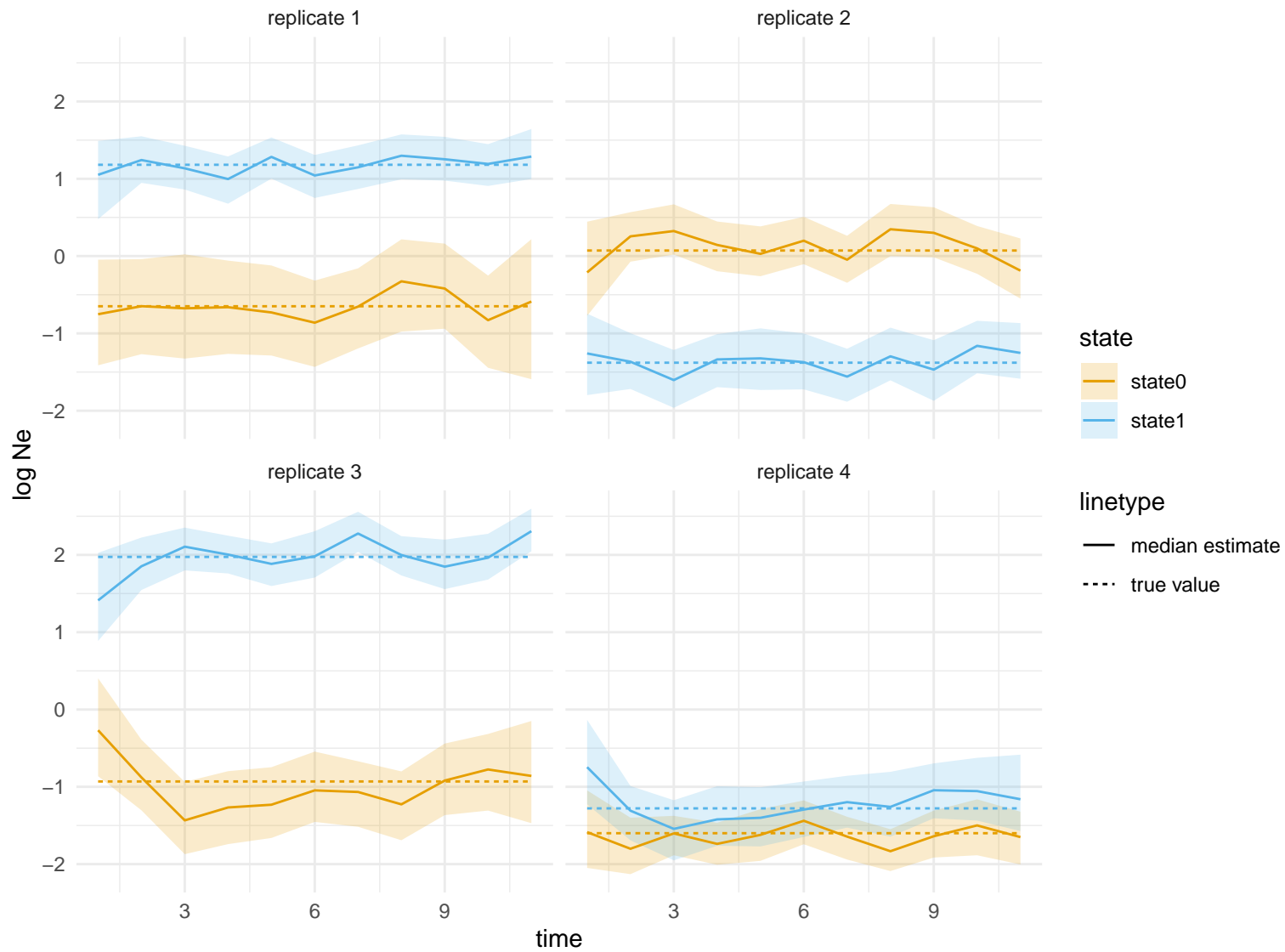

Supplement: S2 Fig — Here, we show how MASCOT-Skyline recovers the effective population sizes when the underlying dynamics are constant. (PDF) [file pcbi.1013421.s002.pdf]

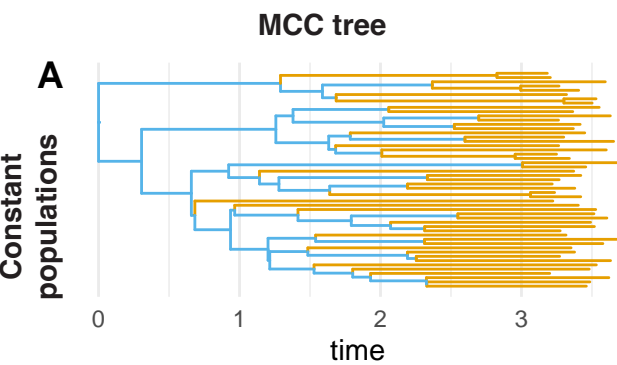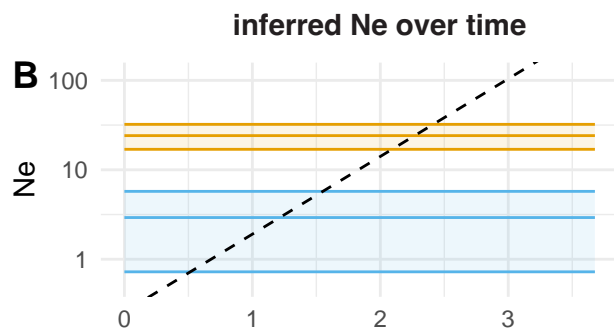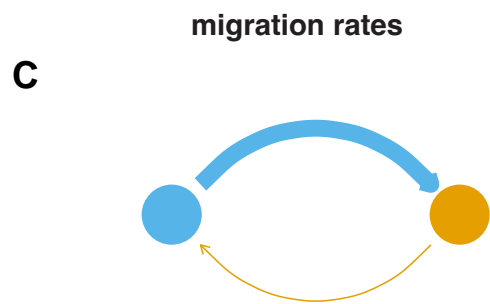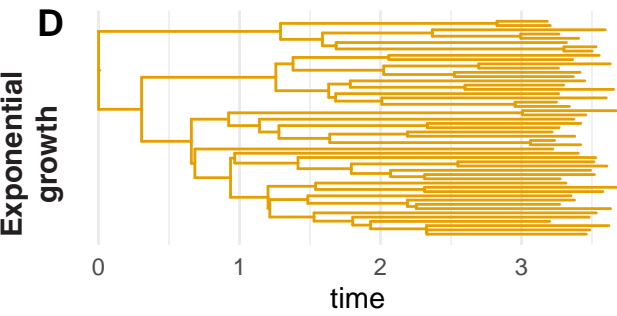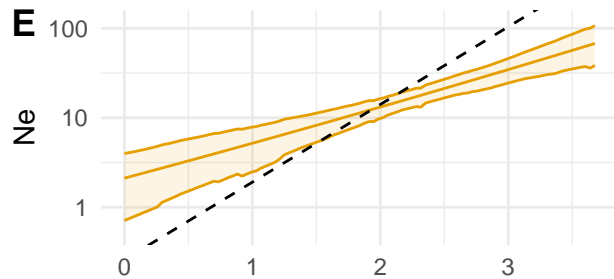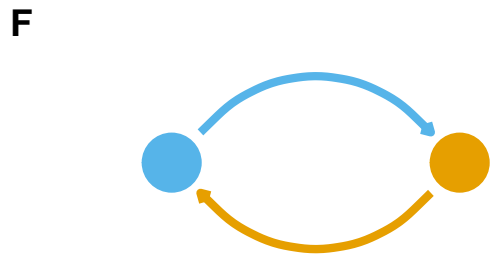

Supplement: S3 Fig — A Inferred node states when assuming a two-state structured coalescent model with two constant populations. B Inferred effective population sizes of the two populations. The dotted black line denotes the true Ne of the orange population. C Inferred migration rates between the two constant populations. D Inferred node states when assuming a two-state structured coalescent model, allowing the two states to grow exponentially. E Inferred effective population sizes over time of the location where all samples were taken from (orange). The Ne of the blue location is sampled under the prior and, therefore, not shown in the figure. F Migration rates between the location where samples were taken and a second (blue) location. (PDF) [file pcbi.1013421.s003.pdf]

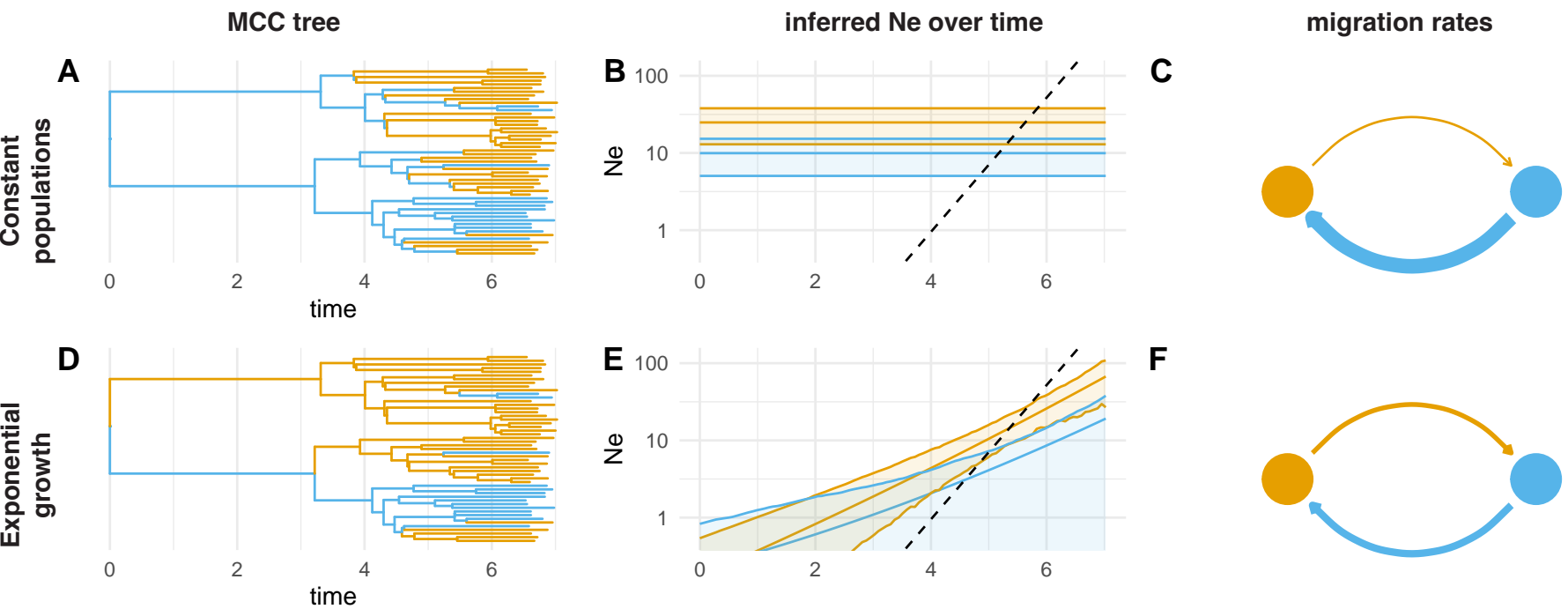

Supplement: S4 Fig — A Inferred node states when assuming a two-state structured coalescent model with two constant populations. B Inferred effective population sizes of the two populations. The dotted line denotes the true Ne of the blue and the orange population. C Inferred migration rates between the two constant populations. D Inferred node states when assuming a two-state structured coalescent model, allowing the two states to grow exponentially. E Inferred effective population sizes over time of the location where all samples were taken from (orange). F Migration rates between the location where samples were taken and a second (blue) location. (PDF) [file pcbi.1013421.s004.pdf]

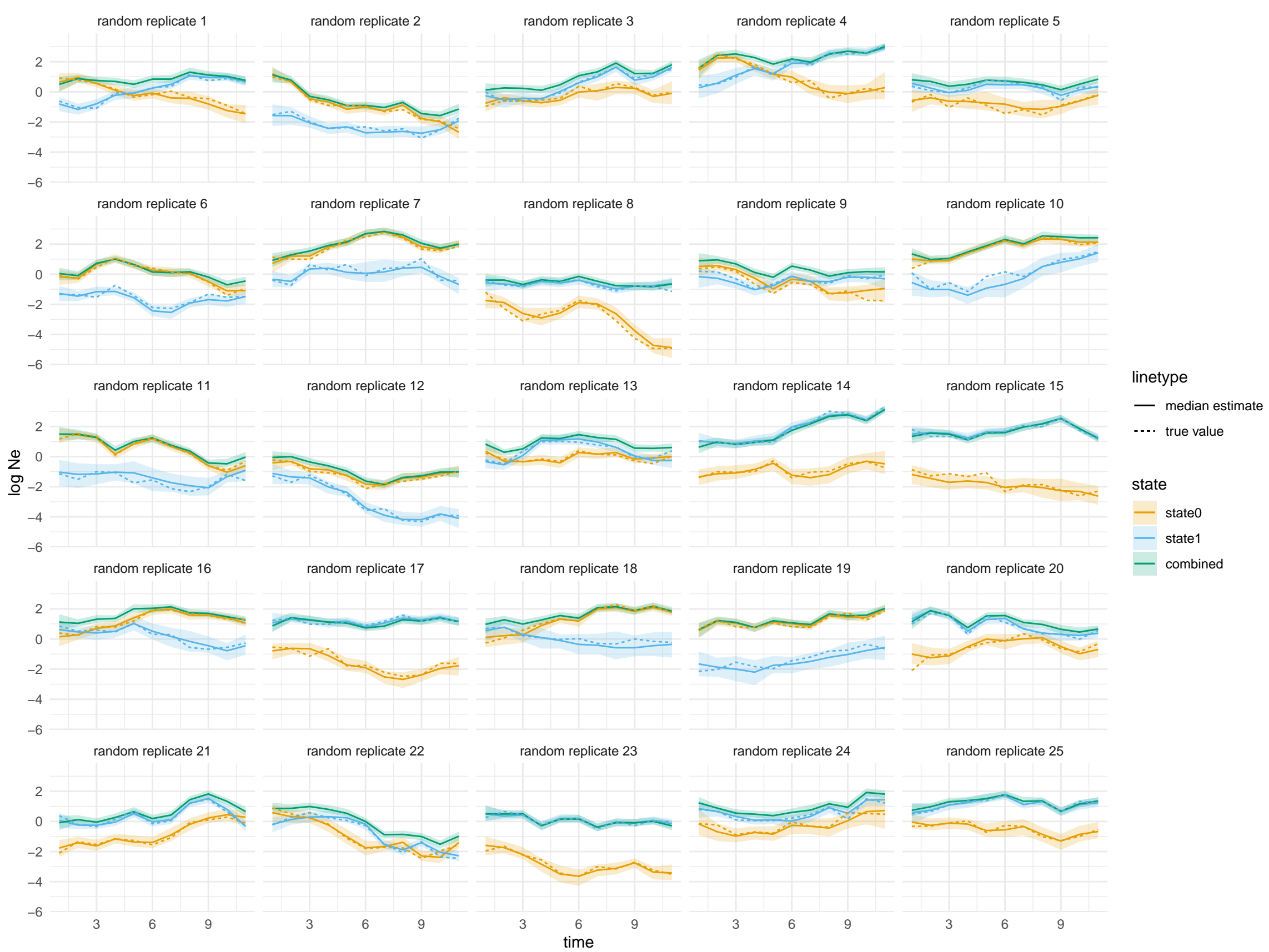

Supplement: S5 Fig — Here, we infer the effective population size (Ne) trajectory for tree simulation under a two-state structured coalescent model with time-varying population size. We do so once modeling the two states (state 0 in orange and state 1 in blue) and once ignoring any population structure (combined in green). (PDF) [file pcbi.1013421.s005.pdf]

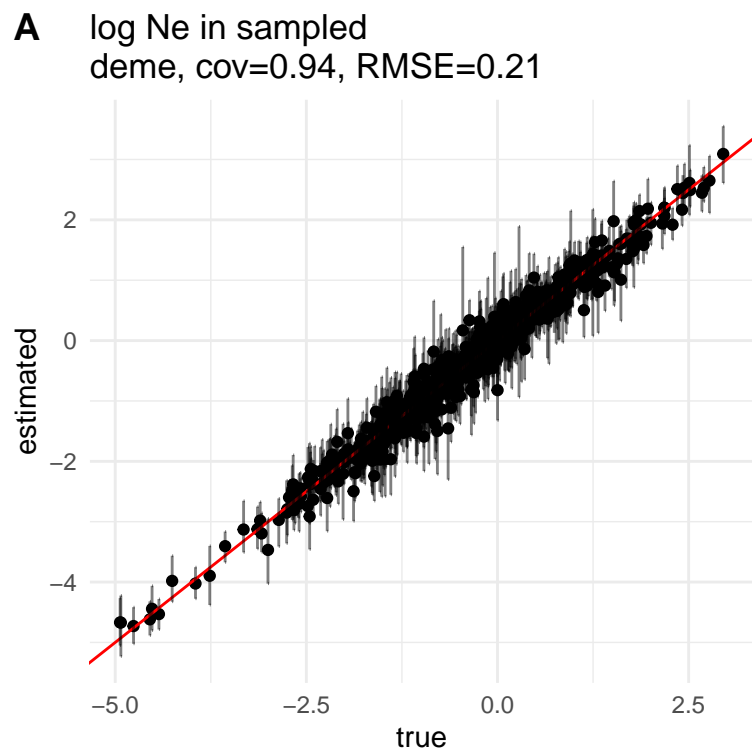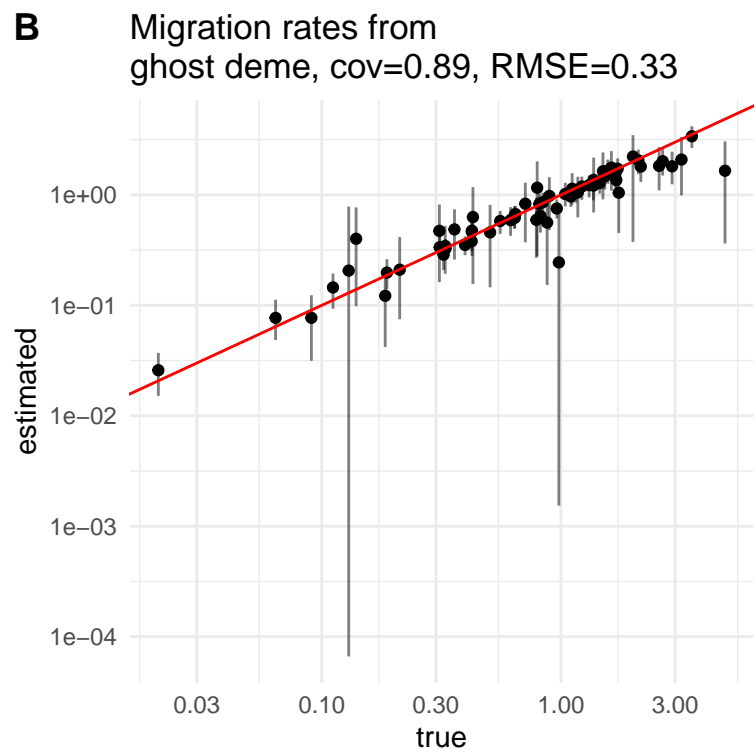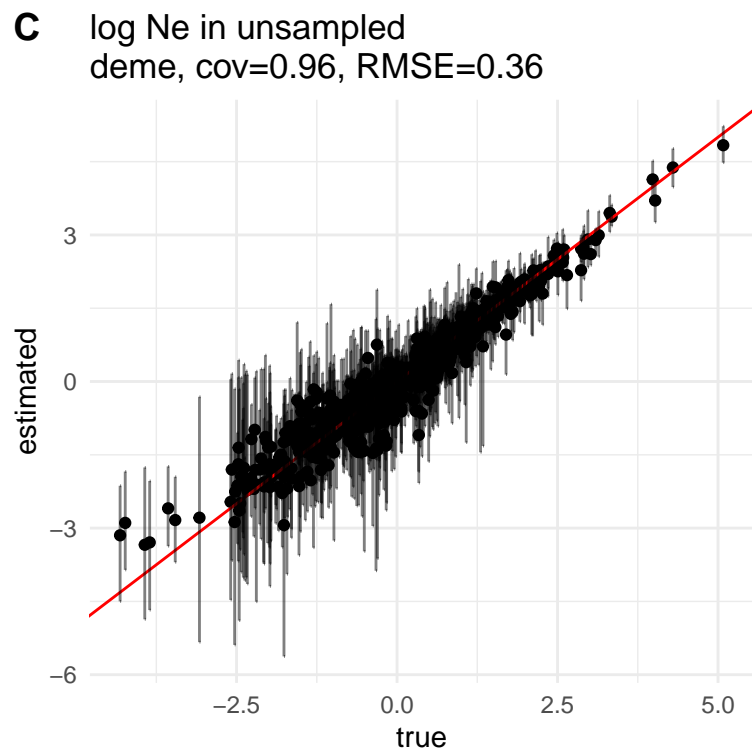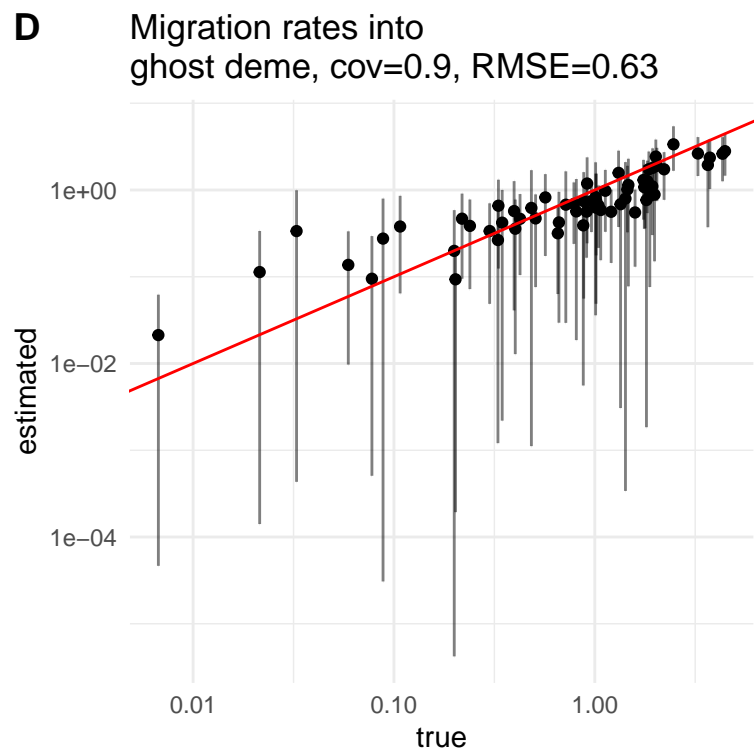

Supplement: S6 Fig — A Inferred vs. true effective population size estimates in the sampled deme. B Inferred vs. true forward in time migration rates from the ghost (unsampled) deme into the sampled deme. C Inferred vs. true effective population size estimates in the ghost deme. B Inferred vs. true forward in time migration rates from sampled deme into the ghost deme. The coverage (cov) of the true value by the 95% highest posterior density interval is shown on the top. (PDF) [file pcbi.1013421.s006.pdf]

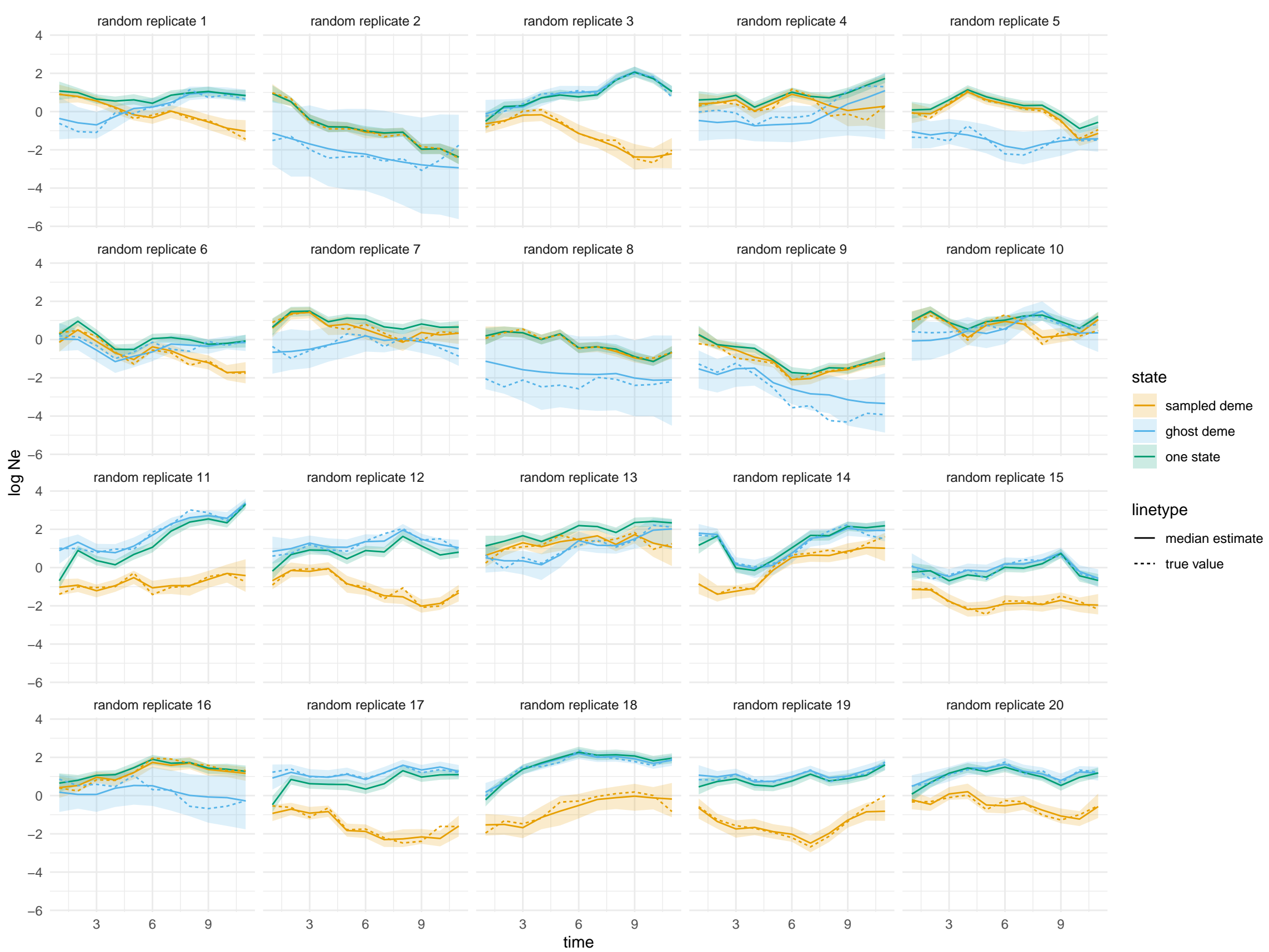

Supplement: S7 Fig — Here, we infer the effective population size (Ne) trajectory for tree simulation under a two-state structured coalescent model with time-varying population size. Only one of those demes (orange) is sampled, while the other one is unsampled (ghost deme). We do so once modeling the two demes (sampled deme in orange and ghost deme in blue) and once ignoring any population structure (in green). The coverages for the Ne’s are computed for all 11 separately estimated, but correlated Ne’s for the 100 simulations. (PDF) [file pcbi.1013421.s007.pdf]

—●— events from source to sink    —●— events sink to source

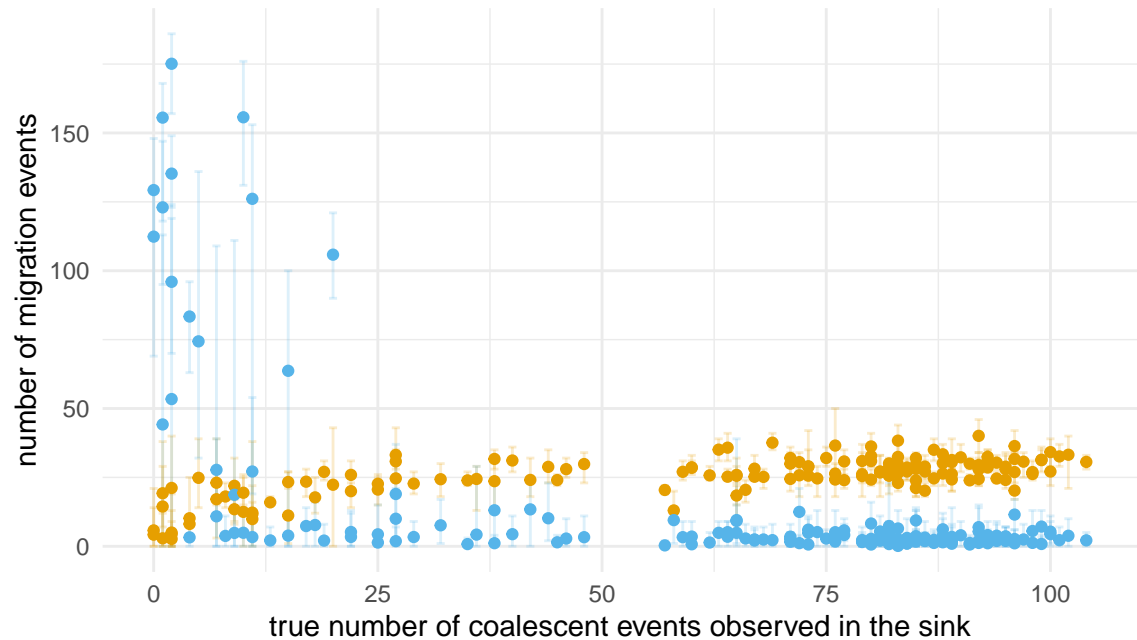

Supplement: S8 Fig — Here, we simulate transmission histories under a 40-state SIR model. The first state is considered the source species and has a population size of 10000 and an R0 of 1.5. States 2 to 20 model localized and oversampled outbreaks in the source species, and each state has a population size of 20 and an R0 of 5. Each of the 20 sink populations has a population size of 5 and an R0 of 10 to model rapid, but contained outbreaks. Transmission, or spillover, can only happen from state 0 to any other state, but not backward. We then vary the amount of sampling in the source species and the sink species. On the x-axis, we show the number of true coalescent events captured in the simulated transmission histories that happened in any of the sink states. On the y-axis, we show the number of inferred migration events from the source to the sink and from the sink to the source. The latter is, in the simulations, always 0, but is inferred to be highly above 0 when there are only a few coalescent events in the sink, and the sink essentially starts to act as a ghost population. (PDF) [file pcbi.1013421.s008.pdf]

—●— events from source to sink    —●— events sink to source

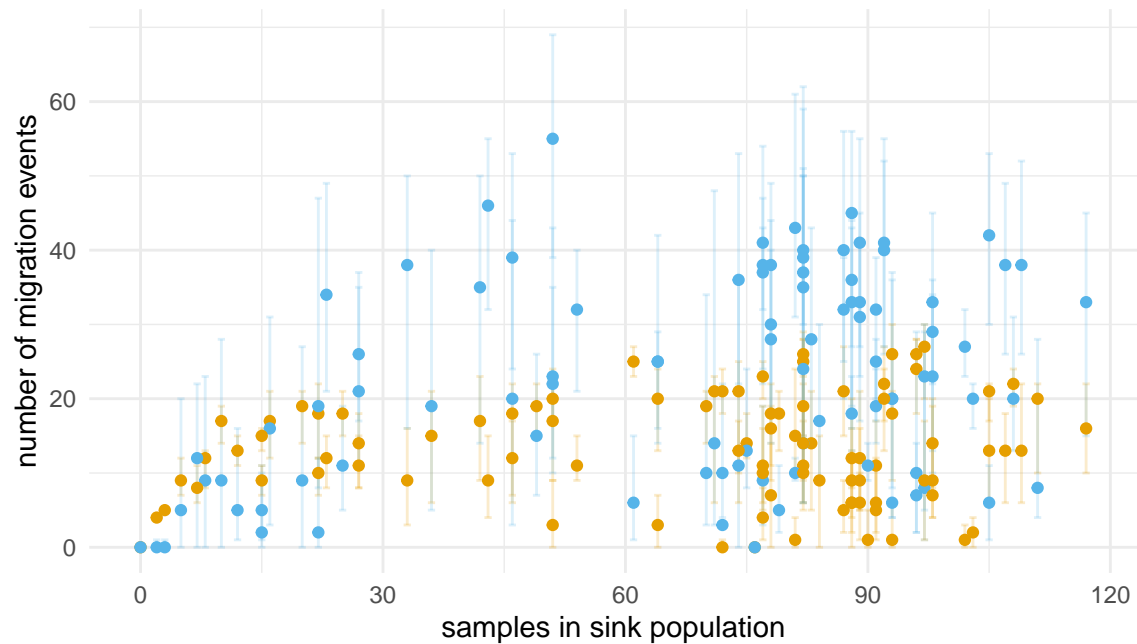

Supplement: S9 Fig — Using the same situations as for S8 Fig, we reconstructed the source sink transmission dynamics using DTA. The y-axis shows the number of inferred transmission events. The true number of transmission events from the sink to the source is 0. The x-axis shows the number of samples in the sink population. (PDF) [file pcbi.1013421.s009.pdf]

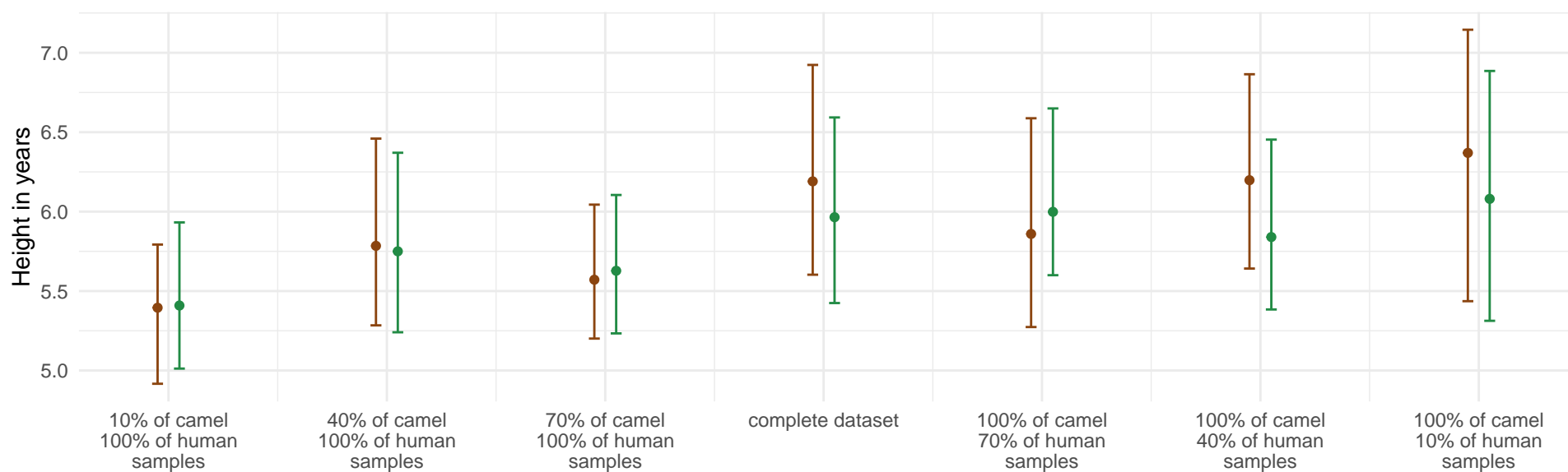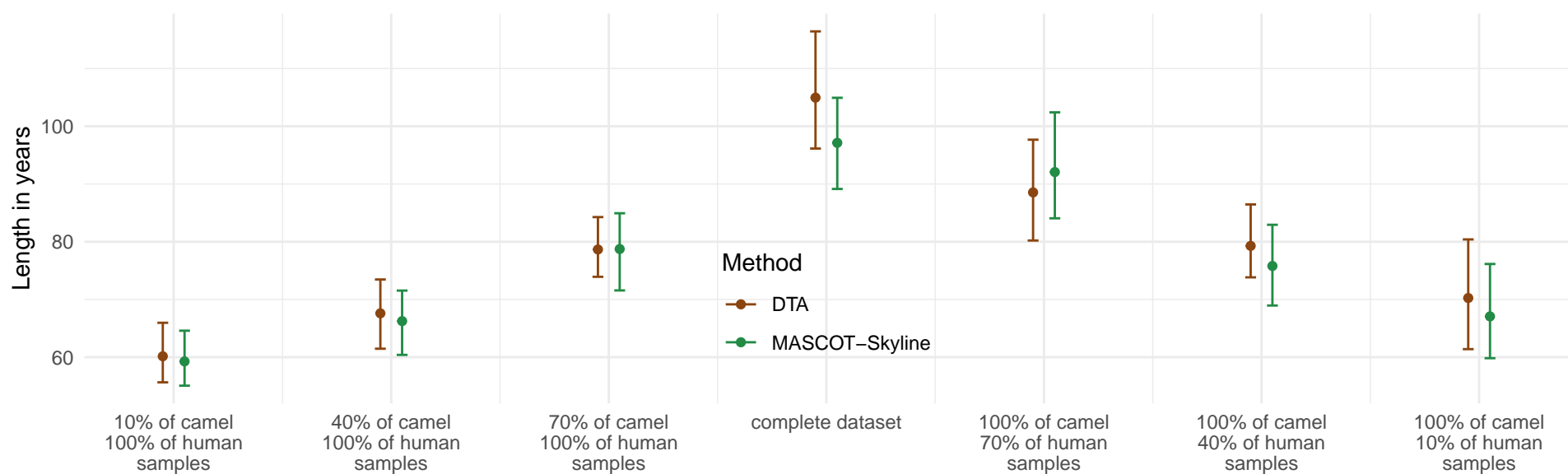

Supplement: S10 Fig — Here, we compare the tree heights and lengths between DTA and MASCOT-Skyline for the different subsampled datasets of the MERS-CoV analysis. The dots denote the median estimate and the error bars the 95% HPD interval. (PDF) [file pcbi.1013421.s010.pdf]

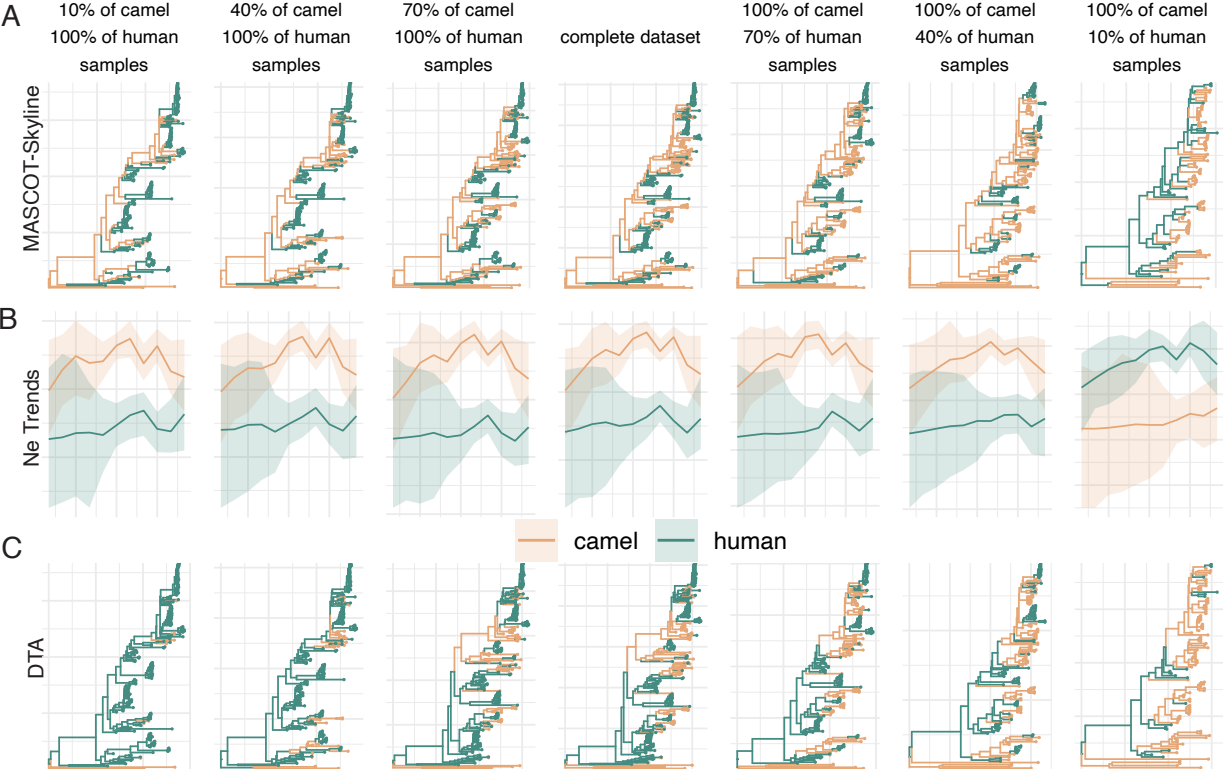

Supplement: S11 Fig — Here, we show the inferences of the transmission dynamics of MERS-CoV between humans and camels when we remove local outbreak clusters in the camel compartment, defined as sequences sampled from the same location in the same month. A Maximum clade credibility (MCC) trees inferred using MASCOT-Skyline for different amounts of samples from camels and humans, from left to right. Each branch is colored by the most likely location of the child node of that branch. B Inferred effective population size trajectories using MASCOT-Skyline for different amounts of samples from camels and humans. C Maximum clade credibility (MCC) trees inferred using DTA. (PDF) [file pcbi.1013421.s011.pdf]

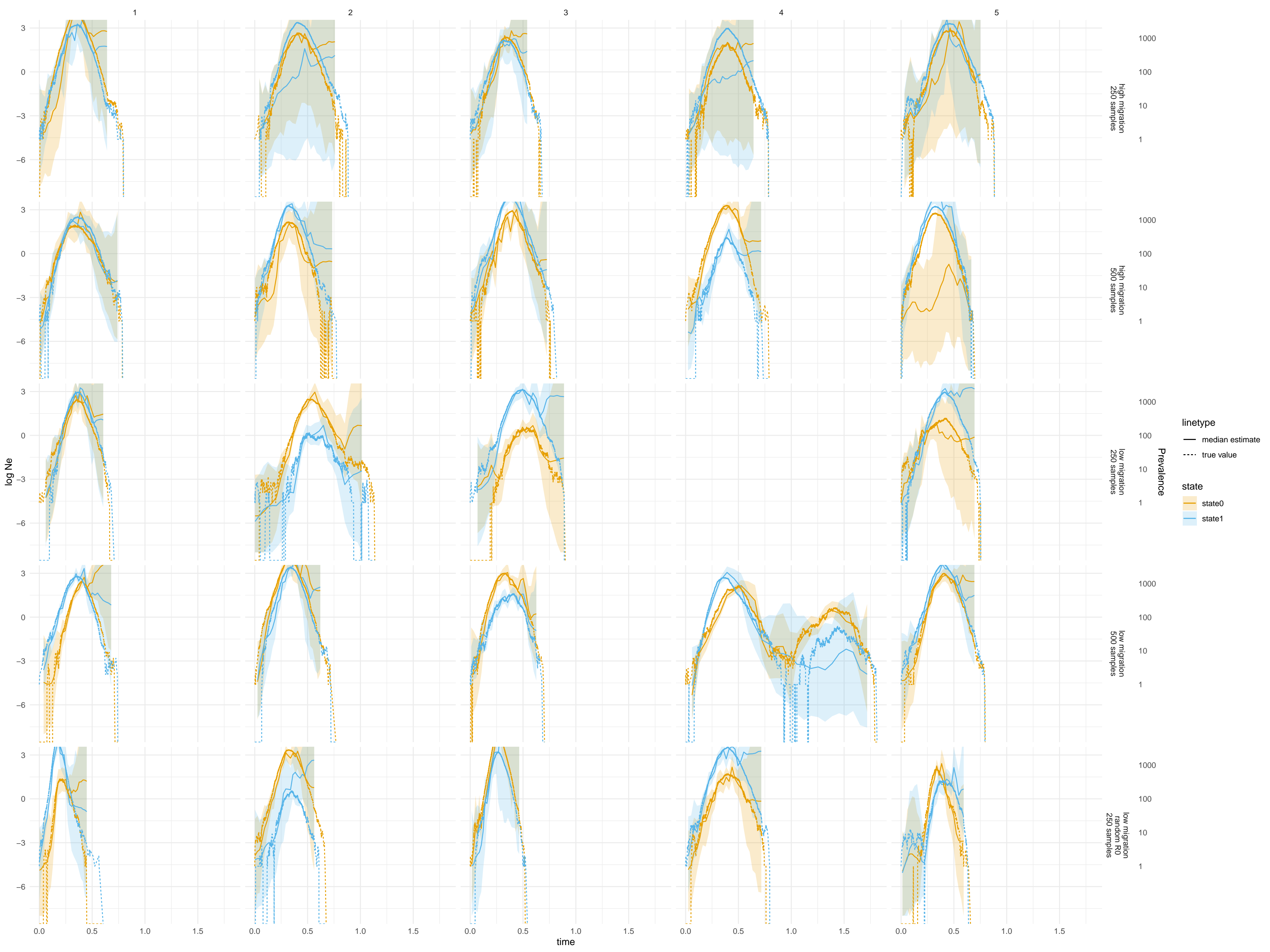

Supplement: S12 Fig — Comparison between simulated prevalences for the two states and the inferred log Ne’s for the two states using MASCOT-Skyline. The trajectories are shown for the first 5 runs of the simulation scenarios, denoted on the left. (PDF) [file pcbi.1013421.s012.pdf]

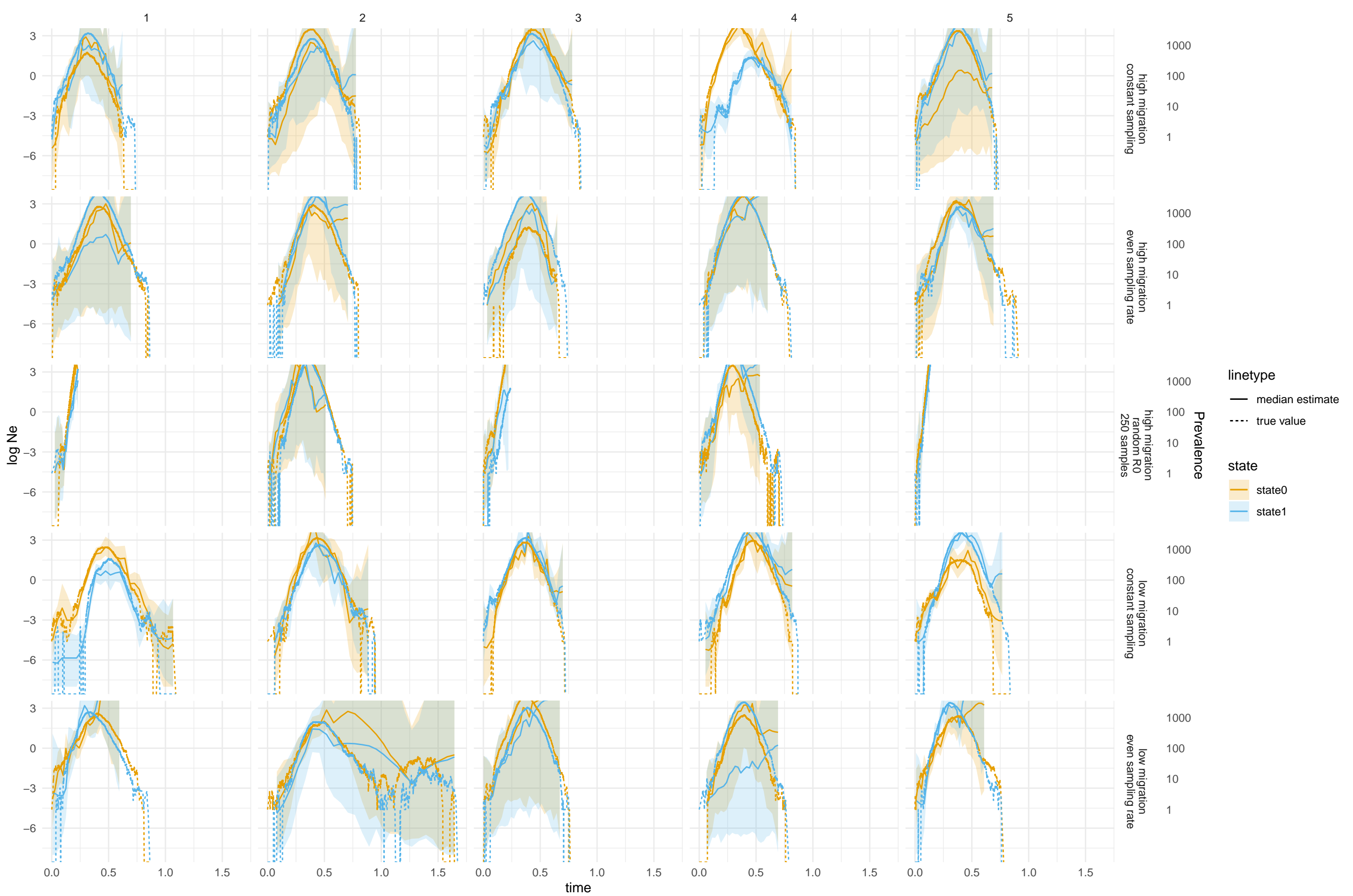

Supplement: S13 Fig — Comparison between simulated prevalences for the two states and the inferred log Ne’s for the two states using MASCOT-Skyline. The trajectories are shown for the first 5 runs of the simulation scenarios, denoted on the left. (PDF) [file pcbi.1013421.s013.pdf]

method DTA MASCOT-Skyline

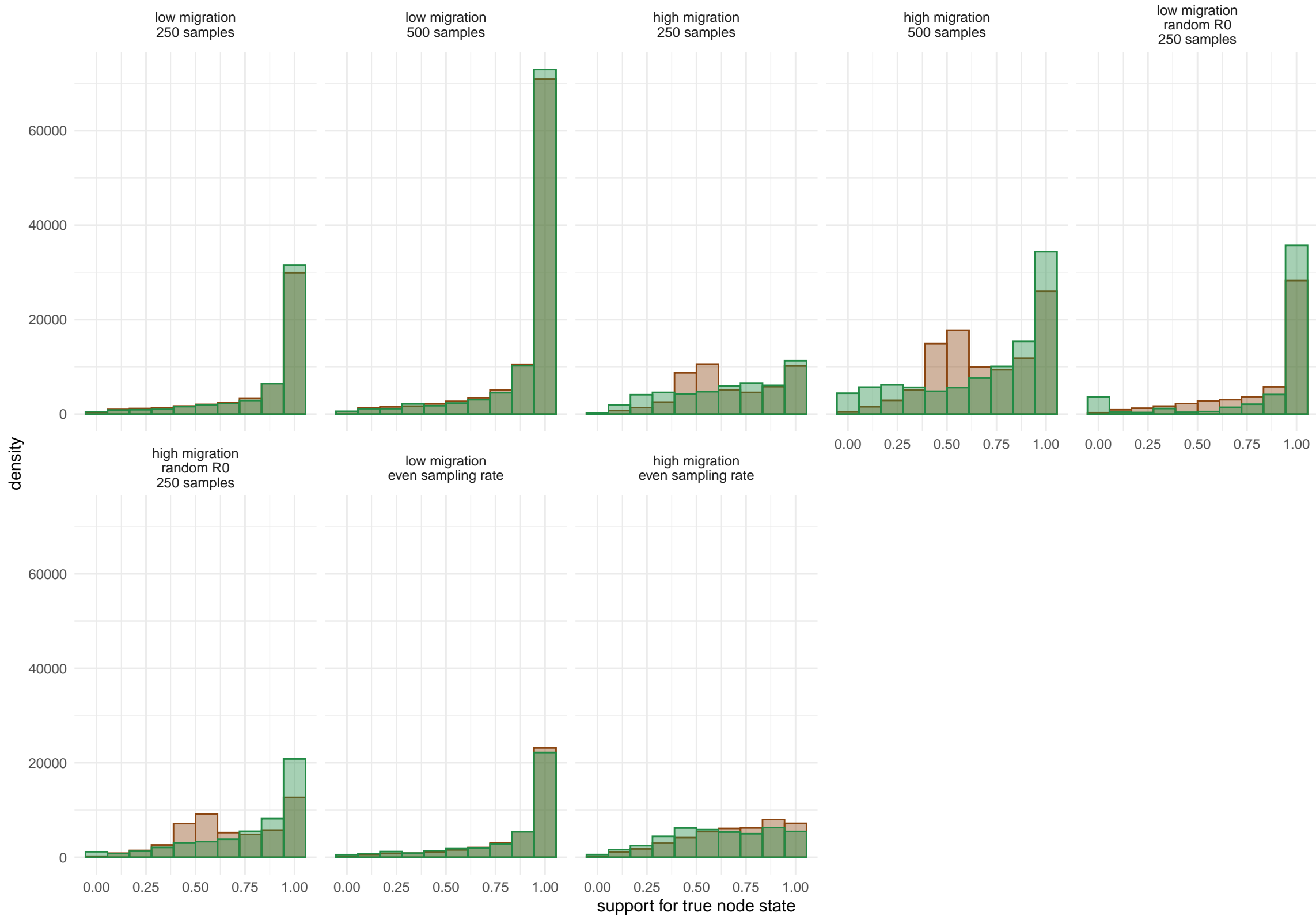

Supplement: S14 Fig — Here, we show the distribution of posterior support for the true node states for the different SIR simulation settings. The posterior node supports are shown DTA and MASCOT-Skyline. Each subplot uses different settings for the simulations: low or high migration rates, where the mean migration rate was 5, respectively. 25. 250 or 500 samples per state, or proportional. (PDF) [file pcbi.1013421.s014.pdf]

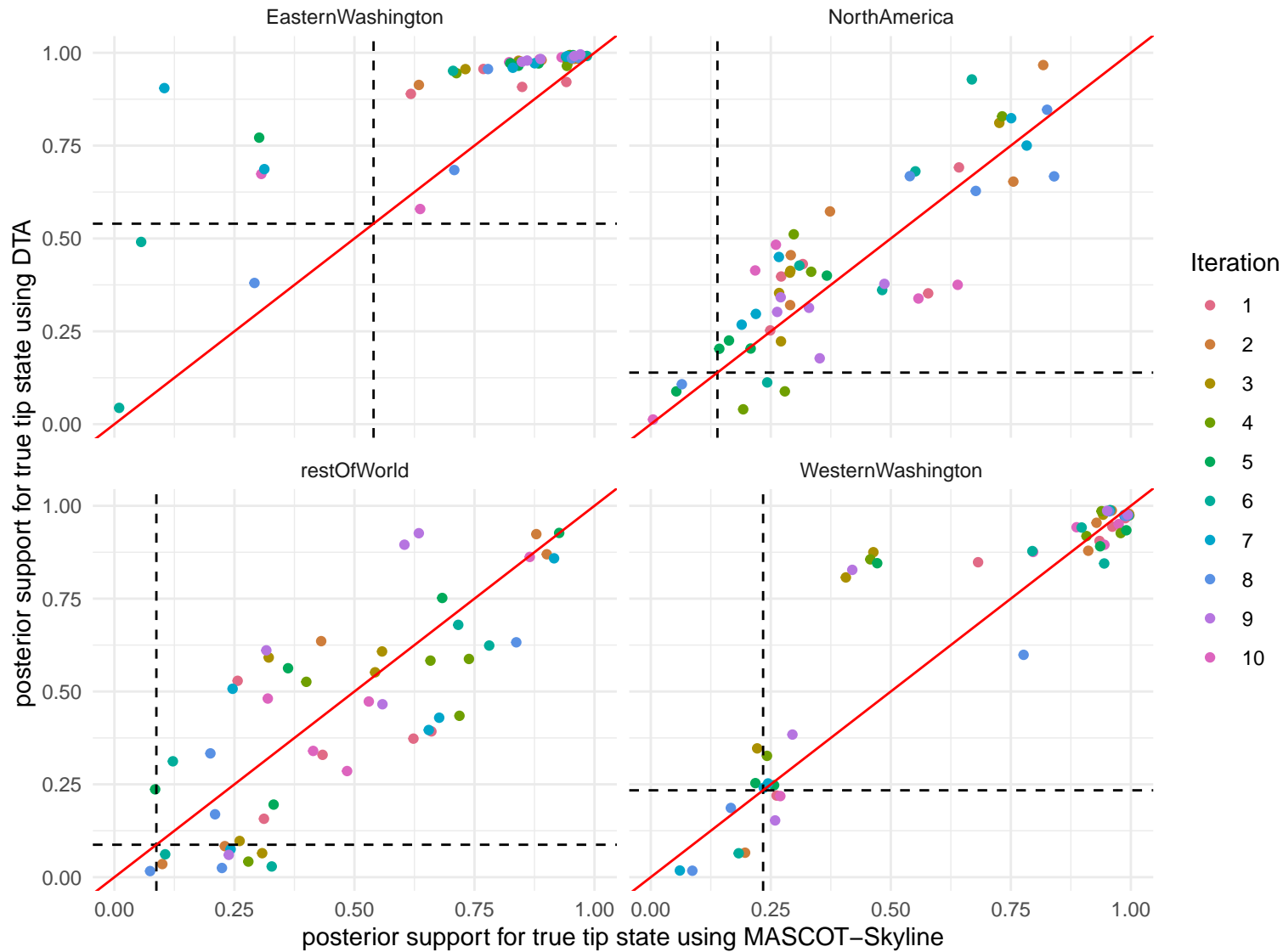

Supplement: S19 Fig — We compare the posterior support for the true sampling location inferred using MASCOT-Skyline and DTA for the four locations in our SARS-CoV-2 dataset. For the inference, the sampling location of random samples in the dataset was masked, and the location was re-inferred. The posterior support for the true location then denotes how much posterior weight the MCMC algorithm is putting on the inferred sampling location between the true sampling location. The dotted lines denote the percentage of samples from each geographic location that is in the analyses, i.e., a line at 0.25 would indicate that 25% of samples in the dataset are from that location. (PDF) [file pcbi.1013421.s019.pdf]

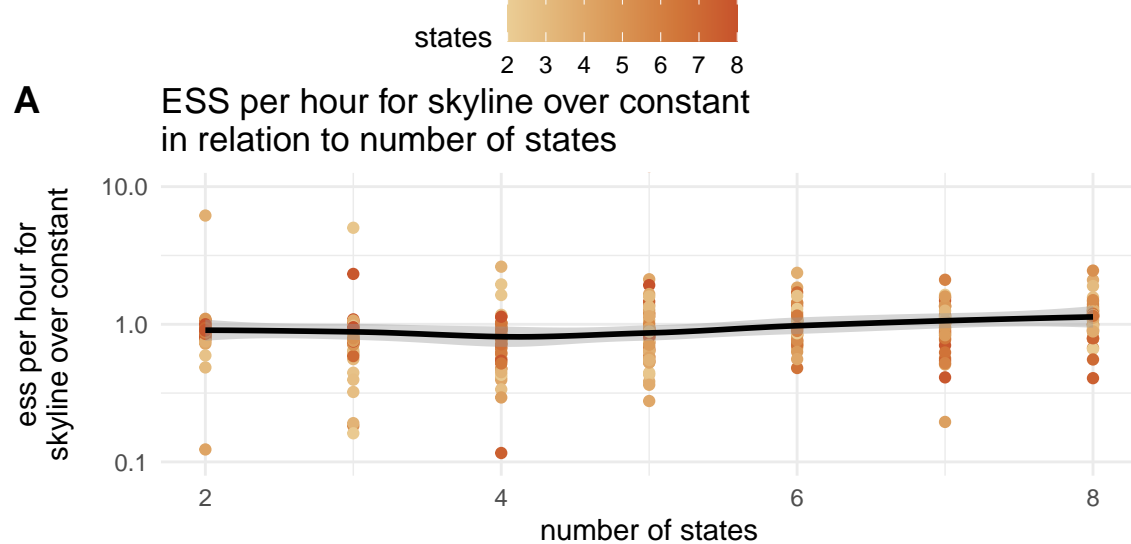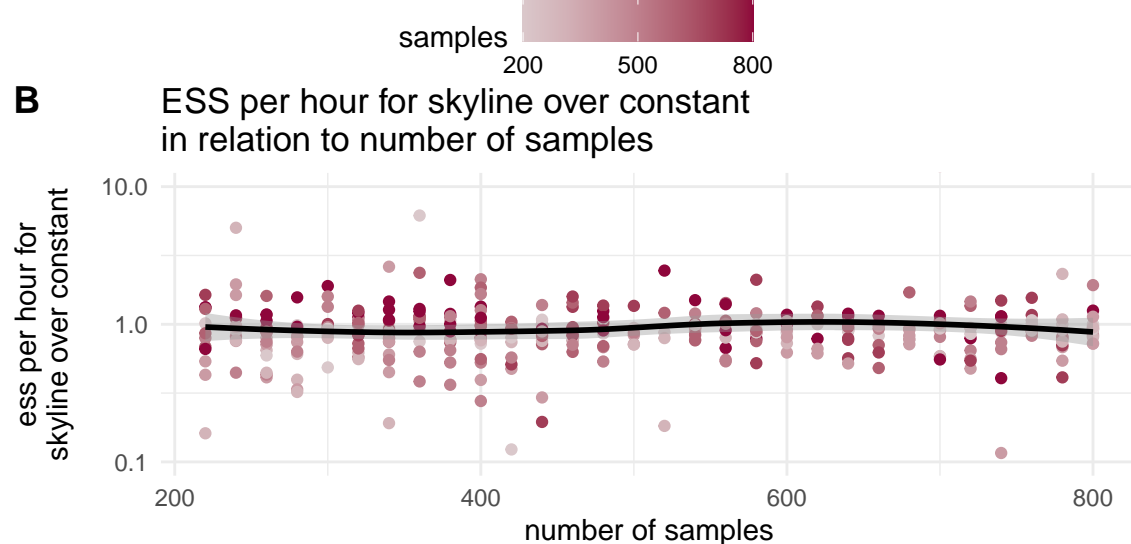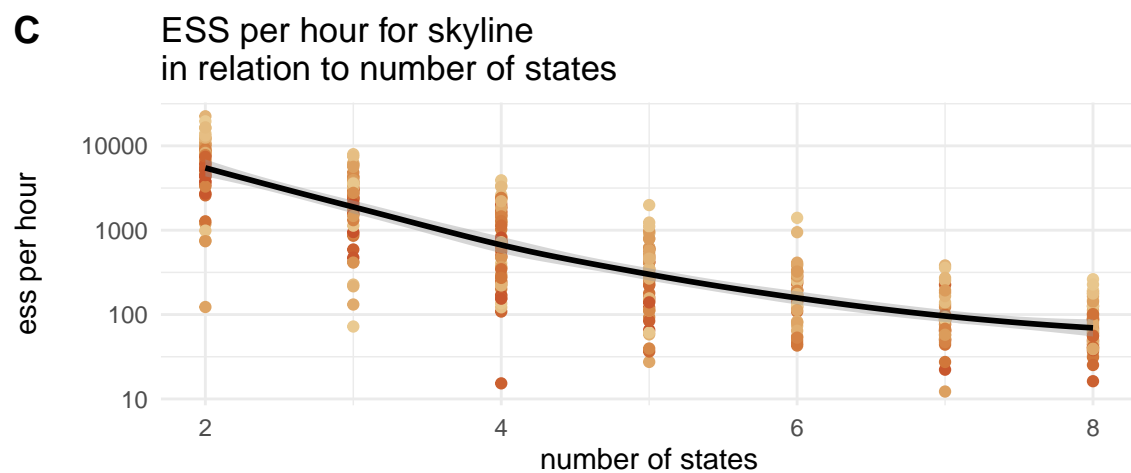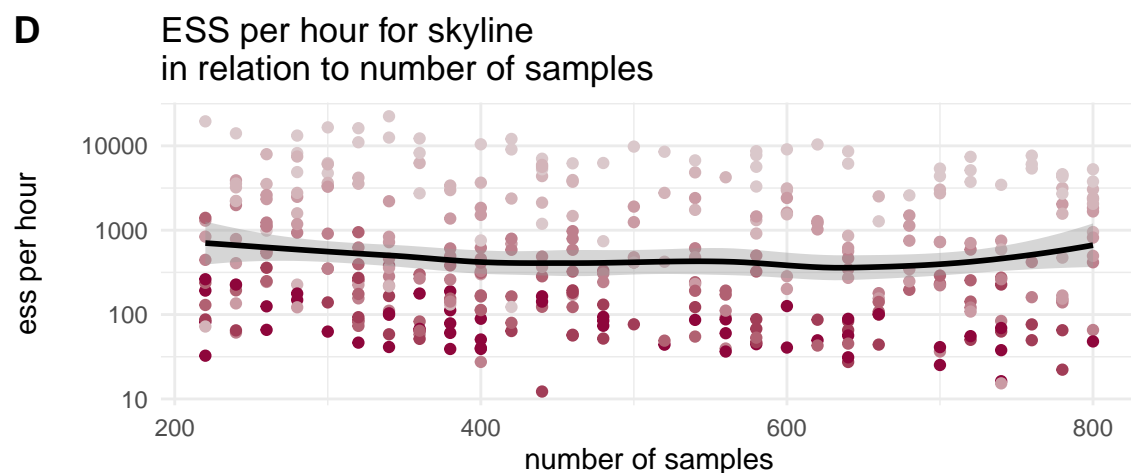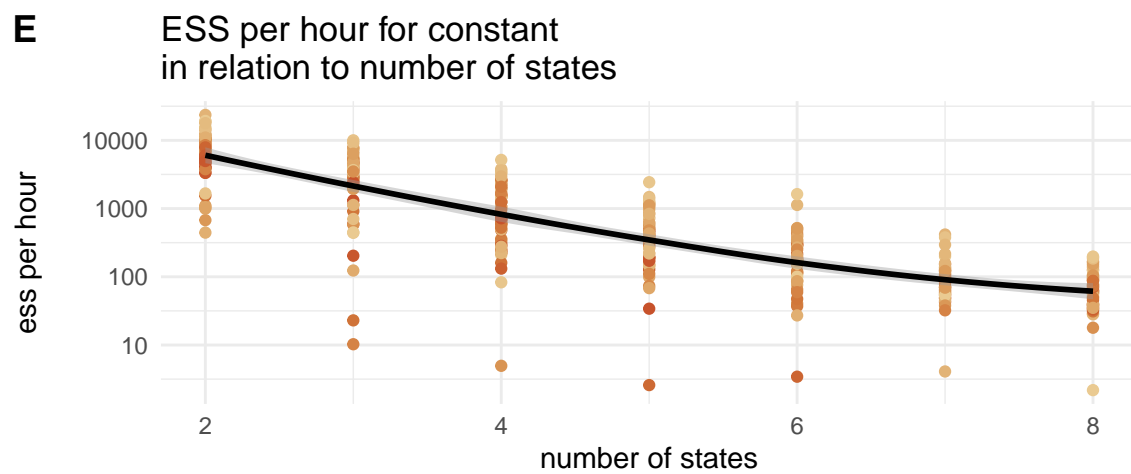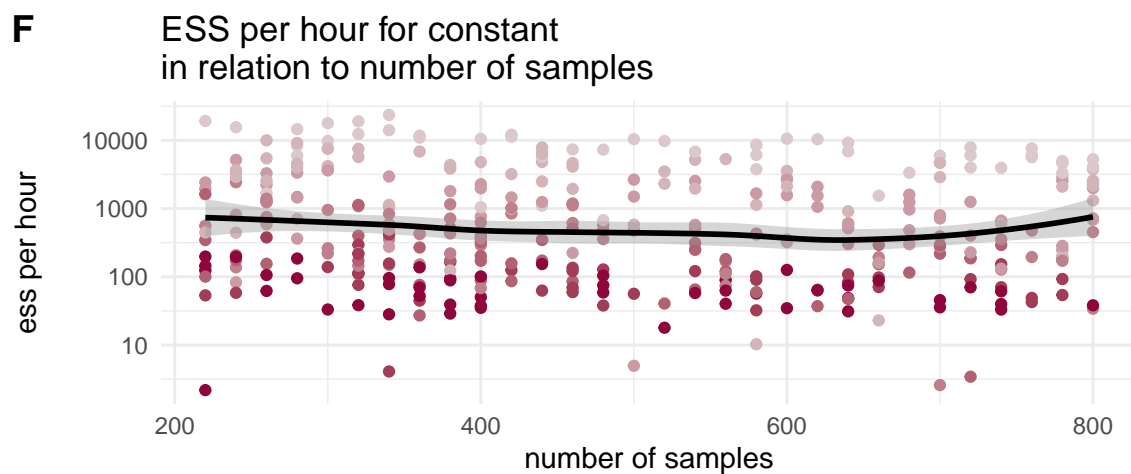

Supplement: S20 Fig — Here, we compare the computational speed of MASCOT-Skyline and MASCOT-Constant for different-sized datasets. To compare the two methods, we simulated phylogenetic trees using a structured coalescent skyline model for different numbers of states and different numbers of samples. We then inferred the population dynamics from the true phylogenetic trees using MASCOT-Skyline and MASCOT-Constant. Next, we plot the ESS per hour of the posterior probability of MASCOT-Skyline over the ESS per hour of the MASCOT-Constant for inference from the same phylogenetic tree (y-axis). The x-axis shows the total number of samples or leaves in the simulation. The different colors show the number of demes in the simulations. A ESSskyline/ESSconstant per hour on a log-scale (y-axis) against the number of demographic states (x-axis). The color of the points indicates the total samples for each run. B ESSskyline/ESSconstant per hour (log-scale) against total samples. The color of the point indicates the number of states. C Absolute ESS per hour under the skyline model (log-scale) vs. number of states. D ESS per hour (skyline model, log-scale) vs. number of samples. E ESS per hour under the constant-population model (log-scale) vs. number of states. F ESS per hour (constant model, log-scale) vs. number of samples. (PDF) [file pcbi.1013421.s020.pdf]

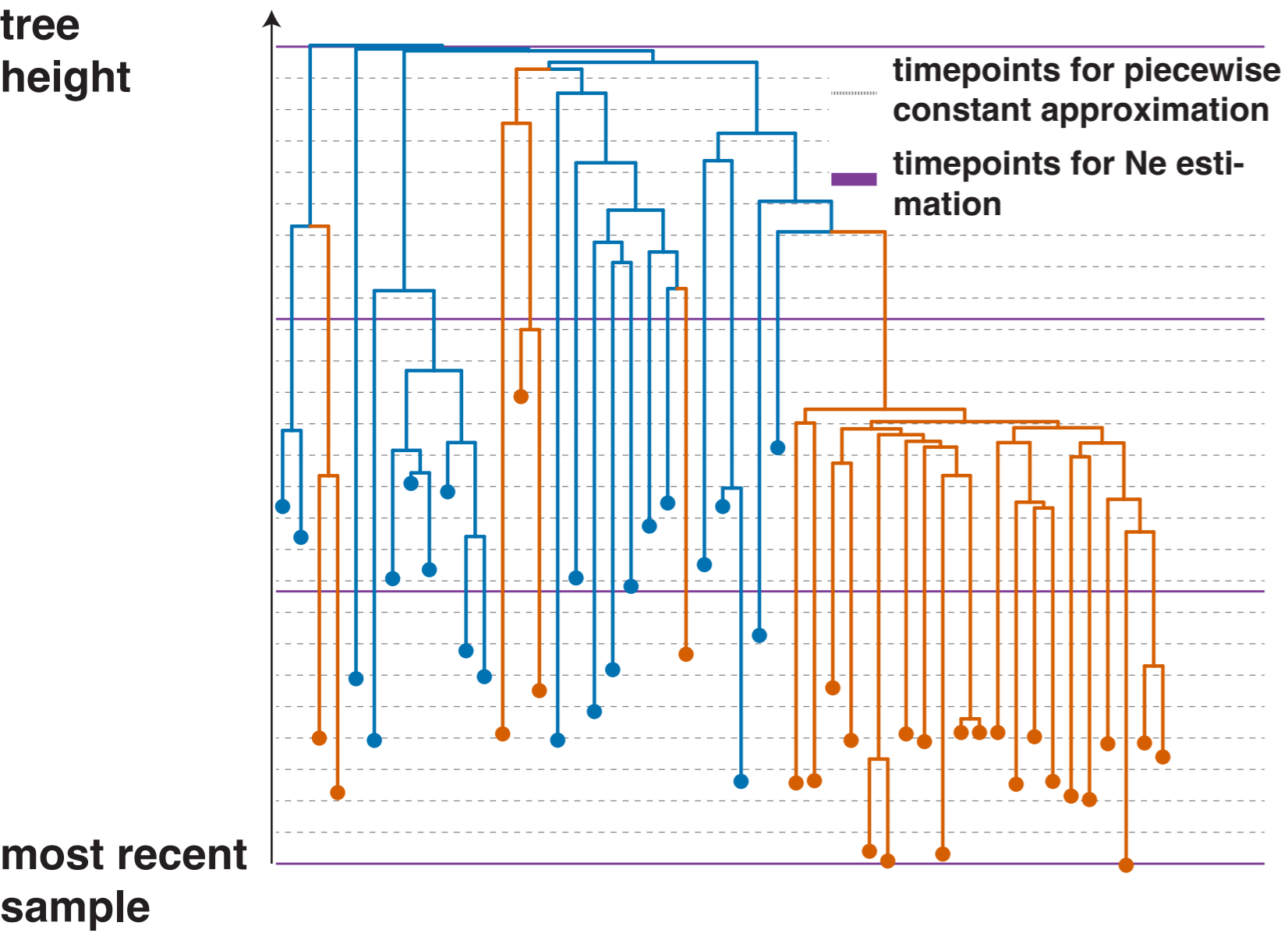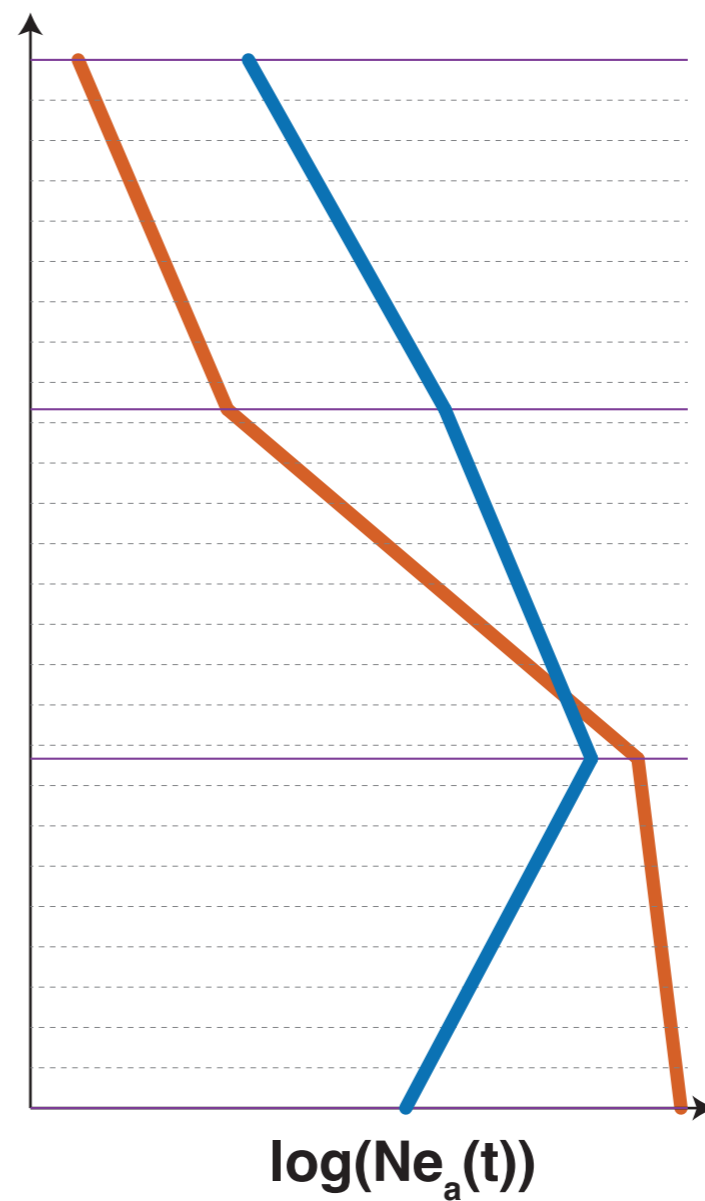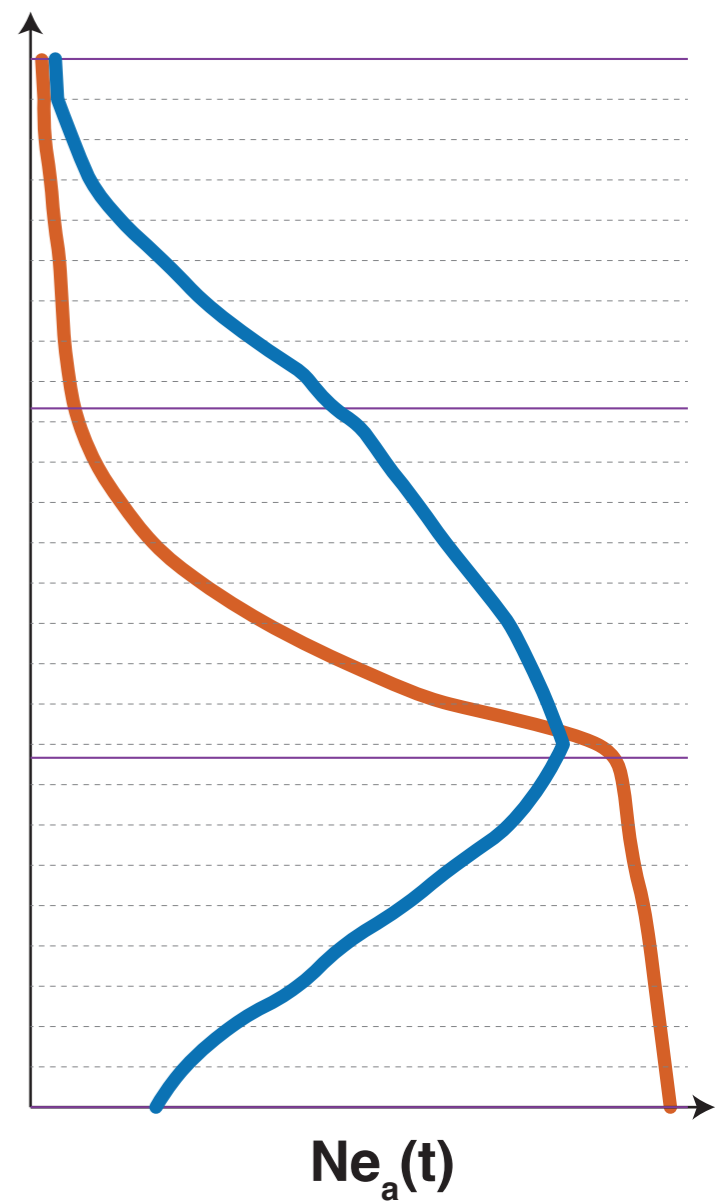

Supplement: S22 Fig — Each location in the dataset has its own population size trajectory. The population size trajectory is considered between the most recent sampled individual (mrsi) and the tree’s root. Here, we consider two more effective population sizes (Ne) between these two points in time. In this case, we estimate four Ne’s per location, with any number of Ne’s possible. Between the four points where we estimate the Ne, we assume that the Ne changes through exponential growth or decline. For the log of the Ne, that means we are using linear interpolation. (PDF) [file pcbi.1013421.s022.pdf]

**A** present log Ne, cov=0.96, RMSE=0.24

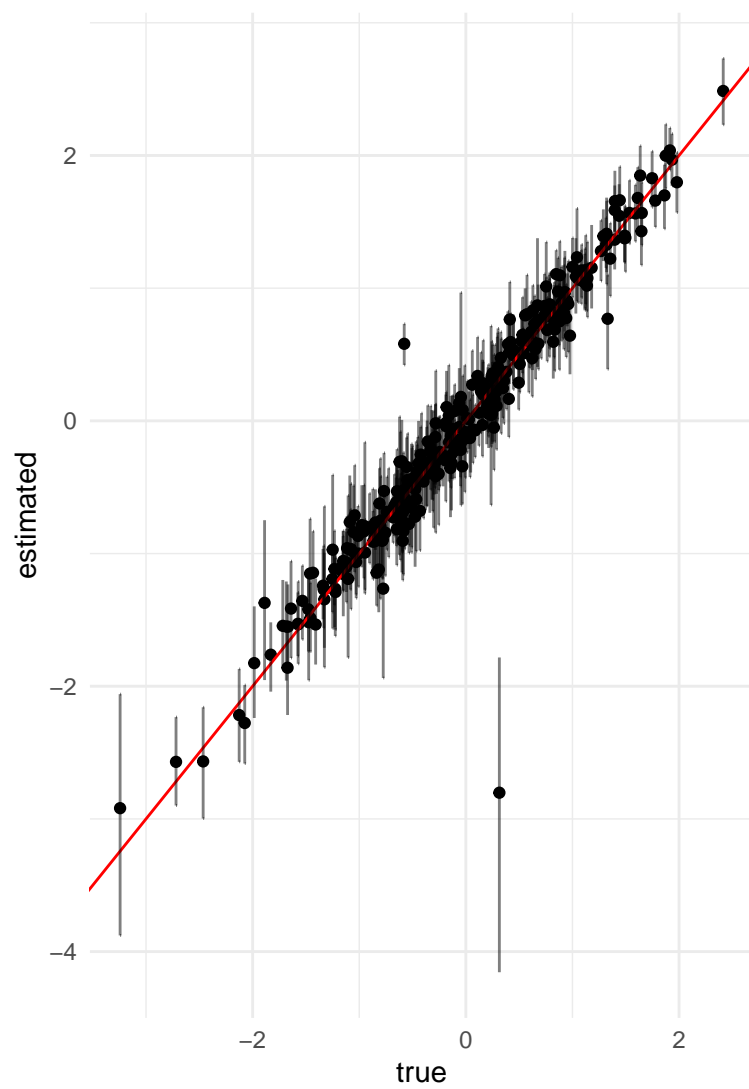

**B** Growth rate, cov= 0.95 , RMSE= 0.08

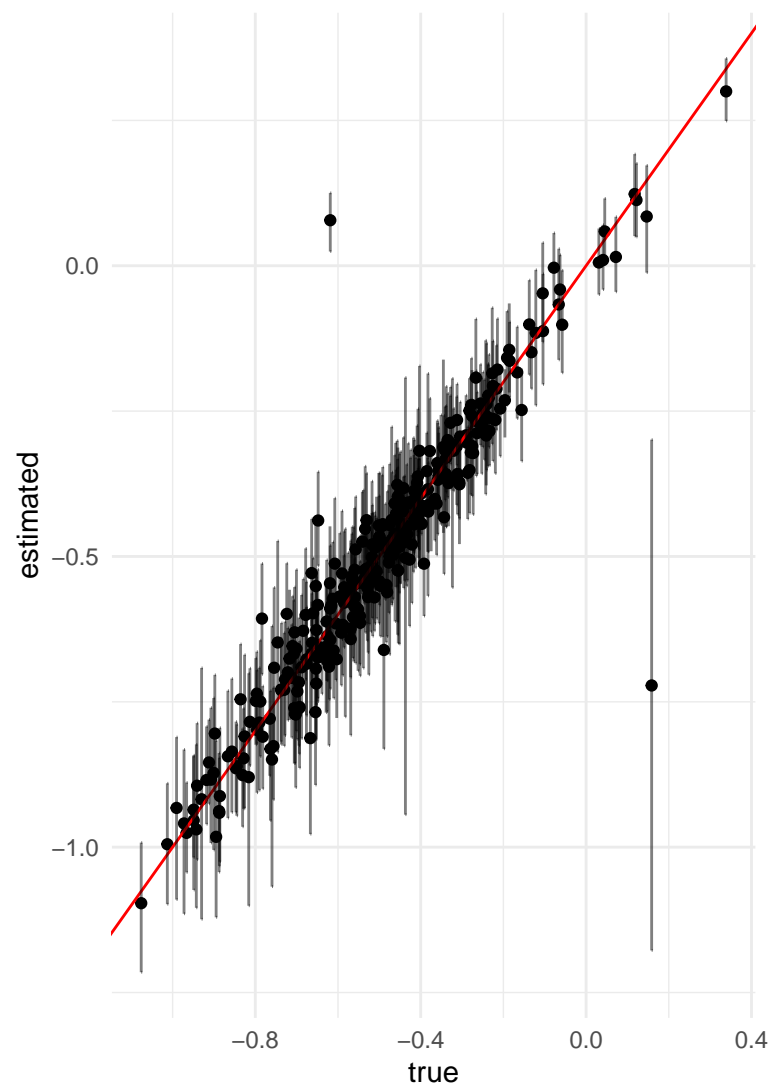

**C** Migration Rates, cov=0.94, RMSE=1.81

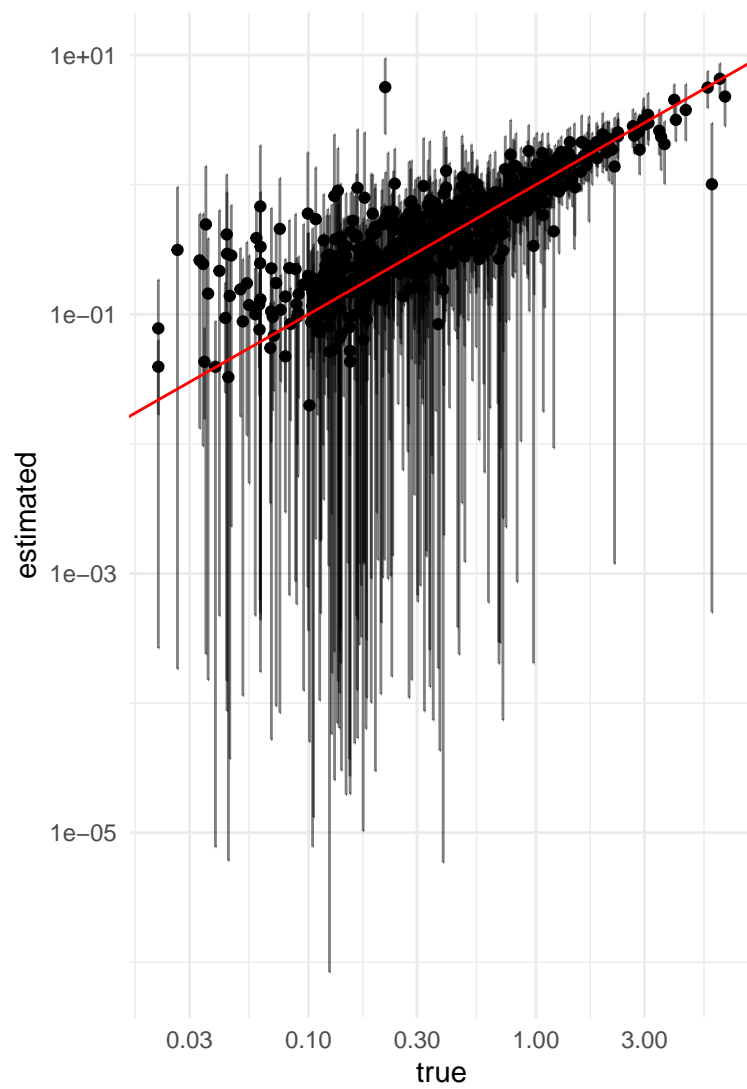

Supplement: S23 Fig — A Inferred vs. true effective log population size at the present. B Inferred vs. true growth rates. C Inferred vs. true forward in time migration rates. The coverage (cov) denotes how often the true, simulated value was part of the 95% highest posterior density intervals. (PDF) [file pcbi.1013421.s023.pdf]
