# Supplementary material for: MASCOT-Skyline integrates population and migration dynamics to enhance phylogeographic reconstructions
Source: PLoS Comput Biol. 2025 Sep 26;21(9):e1013421. doi: 10.1371/journal.pcbi.1013421 (PMC12500135; doi:10.1371/journal.pcbi.1013421)
Supplement: S15 Fig — Here, we show the simulated (x-axis) and estimated (y-axis) migration rates using simulations under a two-state SIR model. The dots show the median estimate, and the error bars show the 95% highest posterior density (HPD) interval. The Pearson correlation coefficients (R) are calculated separately for MASCOT-Skyline and DTA. The coverage of the true value by the 95% HPD is shown after cov. The coefficients are calculated between the simulated values and the median estimates. Each subplot uses different settings for the simulations: low or high migration rates, where the mean migration rate was 5, respectively. 25. 250 or 500 samples per state, or proportional and constant sampling. (PDF) [file pcbi.1013421.s015.pdf]

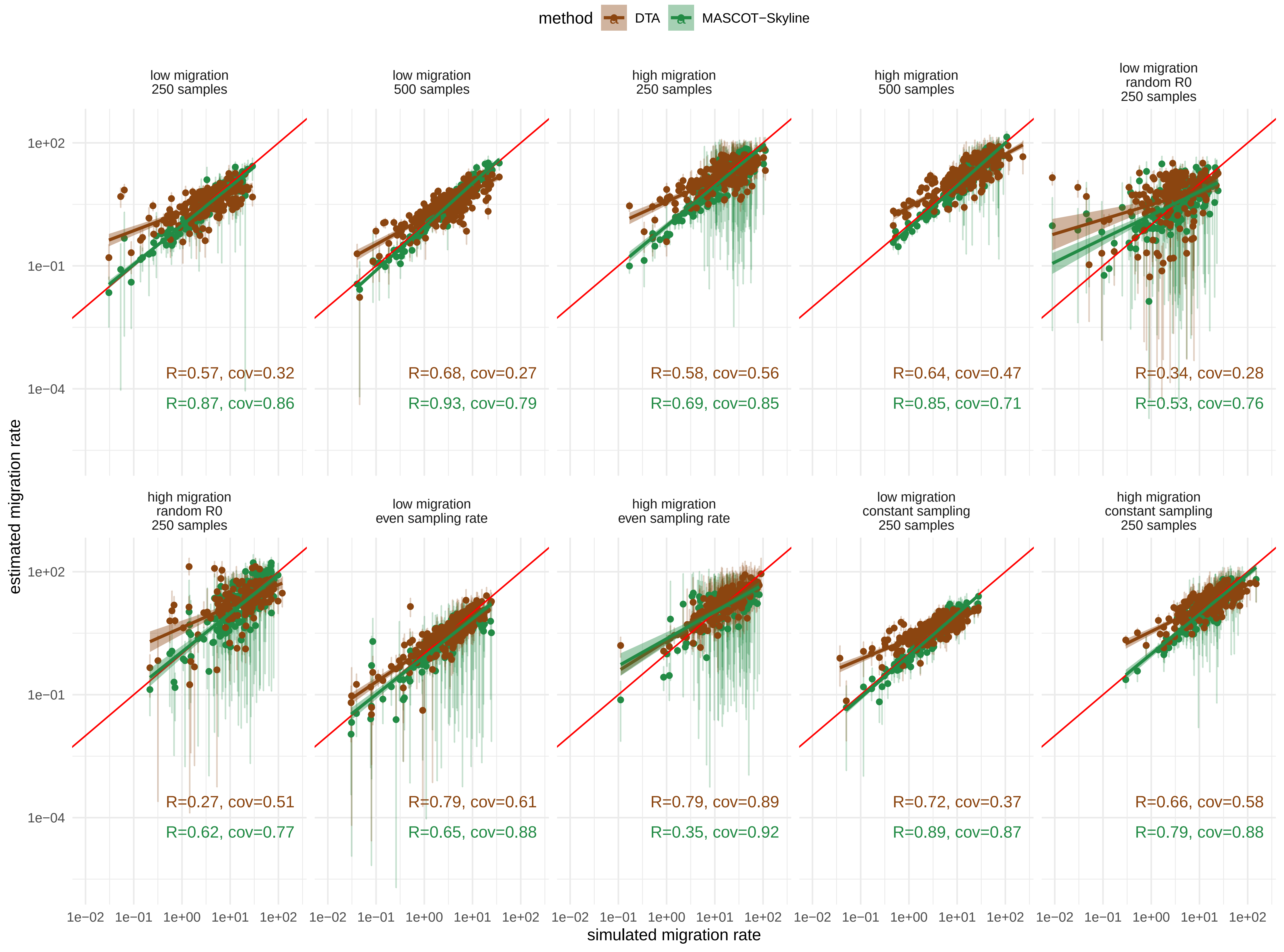
method
DTA
MASCOT−Skyline
low migration
250 samples
R=0.57, cov=0.32
R=0.87, cov=0.86
low migration
500 samples
R=0.68, cov=0.27
R=0.93, cov=0.79
high migration
250 samples
R=0.58, cov=0.56
R=0.69, cov=0.85
high migration
500 samples
R=0.64, cov=0.47
R=0.85, cov=0.71
low migration
random R0
250 samples
R=0.34, cov=0.28
R=0.53, cov=0.76
high migration
random R0
250 samples
R=0.27, cov=0.51
R=0.62, cov=0.77
low migration
even sampling rate
R=0.79, cov=0.61
R=0.65, cov=0.88
high migration
even sampling rate
R=0.79, cov=0.89
R=0.35, cov=0.92
low migration
constant sampling
250 samples
R=0.72, cov=0.37
R=0.89, cov=0.87
high migration
constant sampling
250 samples
R=0.66, cov=0.58
R=0.79, cov=0.88
estimated migration rate
1e+02
1e−01
1e−04
simulated migration rate
1e−02
1e−01
1e+00
1e+01
1e+02
