# Supplementary material for: MASCOT-Skyline integrates population and migration dynamics to enhance phylogeographic reconstructions
Source: PLoS Comput Biol. 2025 Sep 26;21(9):e1013421. doi: 10.1371/journal.pcbi.1013421 (PMC12500135; doi:10.1371/journal.pcbi.1013421)
Supplement: S16 Fig — Here, we show the simulated (x-axis) and estimated (y-axis) migration rates using simulations under a two-state SIR model. The dots show the difference between the upper and lower bounds of the 95% highest posterior density interval divided by the median estimate. The red horizontal line shows the line for the upper and lower bounds of the 95% interval of an exponential distribution used as a prior on the migration rates. (PDF) [file pcbi.1013421.s016.pdf]

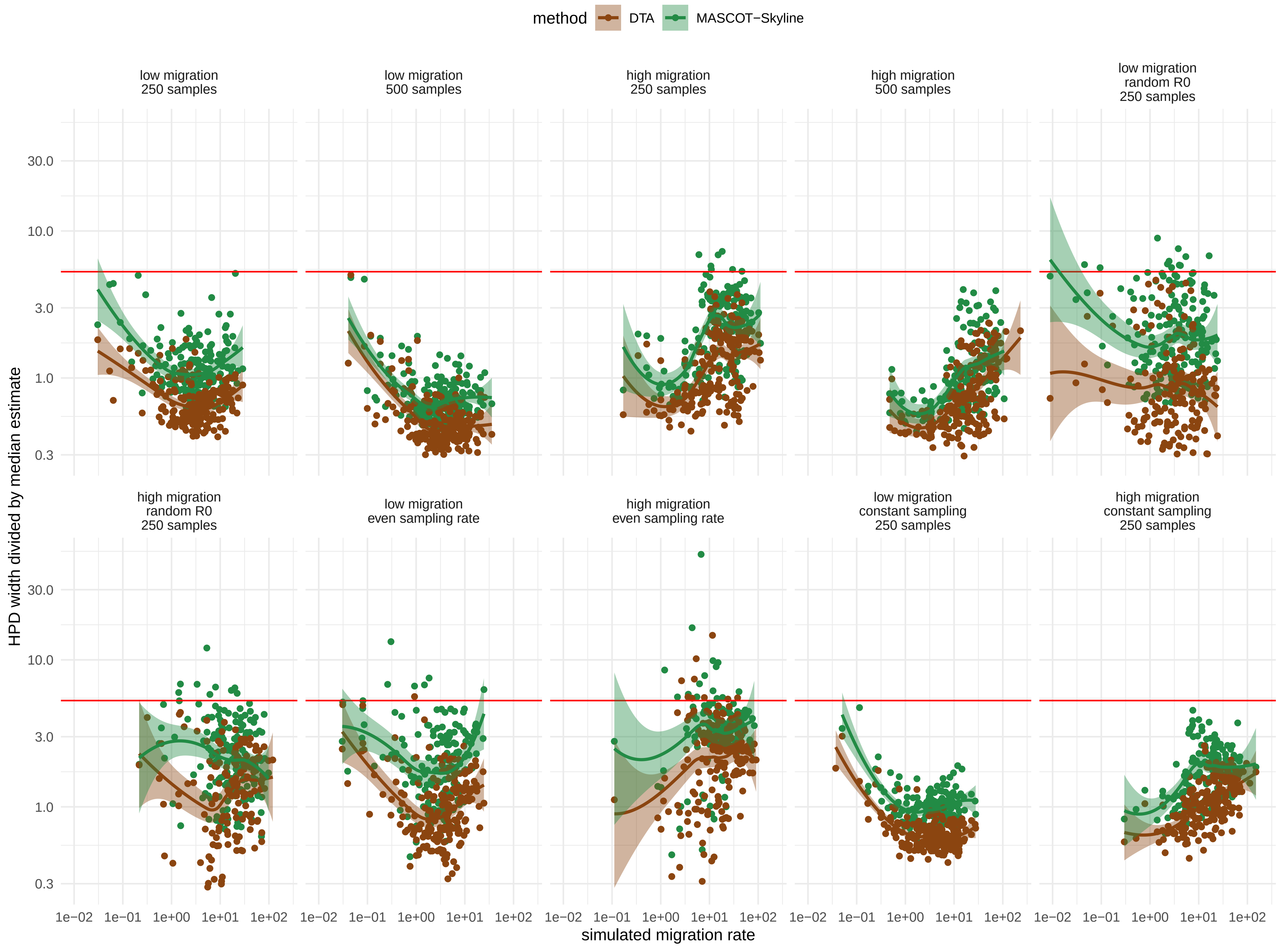

method
DTA
MASCOT−Skyline
low migration
250 samples
low migration
500 samples
high migration
250 samples
high migration
500 samples
low migration
random R0
250 samples
high migration
random R0
250 samples
low migration
even sampling rate
high migration
even sampling rate
low migration
constant sampling
250 samples
high migration
constant sampling
250 samples
30.0
10.0
3.0
1.0
0.3
HPD width divided by median estimate
1e−02
1e−01
1e+00
1e+01
1e+02
simulated migration rate
