# Supplementary material for: MASCOT-Skyline integrates population and migration dynamics to enhance phylogeographic reconstructions
Source: PLoS Comput Biol. 2025 Sep 26;21(9):e1013421. doi: 10.1371/journal.pcbi.1013421 (PMC12500135; doi:10.1371/journal.pcbi.1013421)
Supplement: S17 Fig — Here, we show the simulated (x-axis) and estimated (y-axis) migration rates using simulations under a two-state SIR model. The dots show the median estimate, and the error bars show the 95% highest posterior density (HPD) interval. The Pearson correlation coefficients (R) are calculated independently for MASCOT-Skyline and DTA, are shown in the top left corner of each plot, and are computed between the log of the true value and the log of the median estimate. We additionally show how often the 95% HPD interval covers the true value (cov). The coefficients are calculated between the simulated values and the median estimates. Each subplot uses different settings for the simulations, i.e., low or high migration rates, where the mean migration rate was 5 resp. 25. 250 or 500 samples per state, or proportional and constant sampling. (PDF) [file pcbi.1013421.s017.pdf]

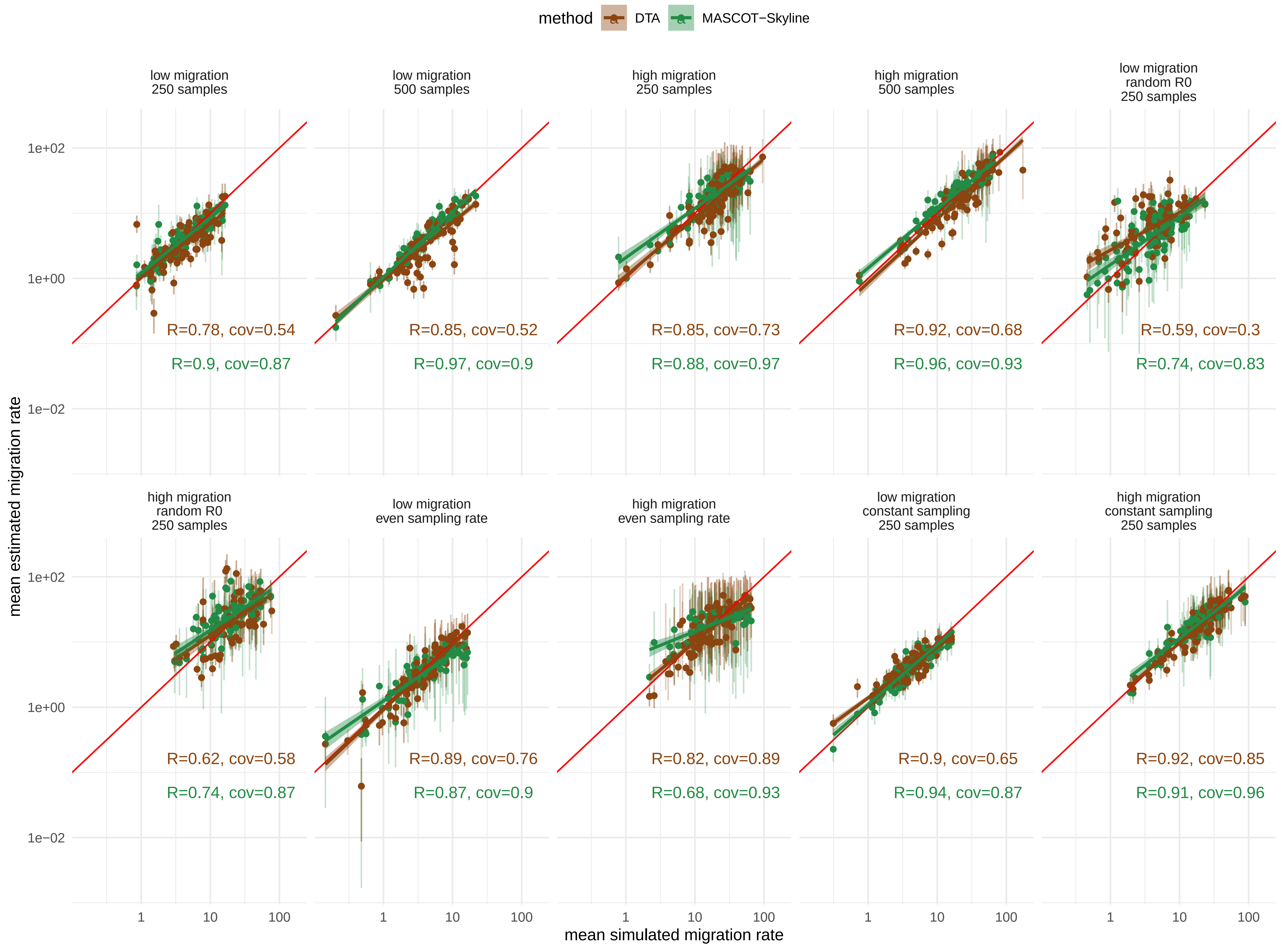

method
DTA
MASCOT−Skyline
low migration 250 samples
low migration 500 samples
high migration 250 samples
high migration 500 samples
low migration random R0 250 samples
high migration random R0 250 samples
low migration even sampling rate
high migration even sampling rate
low migration constant sampling 250 samples
high migration constant sampling 250 samples
R=0.78, cov=0.54
R=0.9, cov=0.87
R=0.85, cov=0.52
R=0.97, cov=0.9
R=0.85, cov=0.73
R=0.88, cov=0.97
R=0.92, cov=0.68
R=0.96, cov=0.93
R=0.59, cov=0.3
R=0.74, cov=0.83
R=0.62, cov=0.58
R=0.74, cov=0.87
R=0.89, cov=0.76
R=0.87, cov=0.9
R=0.82, cov=0.89
R=0.68, cov=0.93
R=0.9, cov=0.65
R=0.94, cov=0.87
R=0.92, cov=0.85
R=0.91, cov=0.96
1e+02
1e+00
1e−02
1
10
100
mean estimated migration rate
mean simulated migration rate
