# Supplementary material for: MASCOT-Skyline integrates population and migration dynamics to enhance phylogeographic reconstructions
Source: PLoS Comput Biol. 2025 Sep 26;21(9):e1013421. doi: 10.1371/journal.pcbi.1013421 (PMC12500135; doi:10.1371/journal.pcbi.1013421)
Supplement: S18 Fig — Here, we compare the simulated migration rate ratio to the estimated ratio of migration rates between the two states in the SIR model. The migration rate estimates are shown for DTA, MASCOT-Skyline, and DTA with case correction, where we multiply the ratio of migration rates by the ratio of cumulative incidence over the simulations to correct for differences in population size. The dots show the median estimate of the migration ratios, and the error bars show the 95% highest posterior density (HPD) interval. The Pearson correlation coefficients (R) are calculated independently for MASCOT-Skyline and DTA and DTA with case correction. The correlation coefficients are computed between the log of the true value and the log of the median estimate. We additionally show how often the 95% HPD interval covers the true value (cov). Each subplot uses different settings for the simulations, that is, low or high migration rates, where the mean migration rate was 5, respectively. 25. 250 or 500 samples per state, proportional, and constant sampling. (PDF) [file pcbi.1013421.s018.pdf]

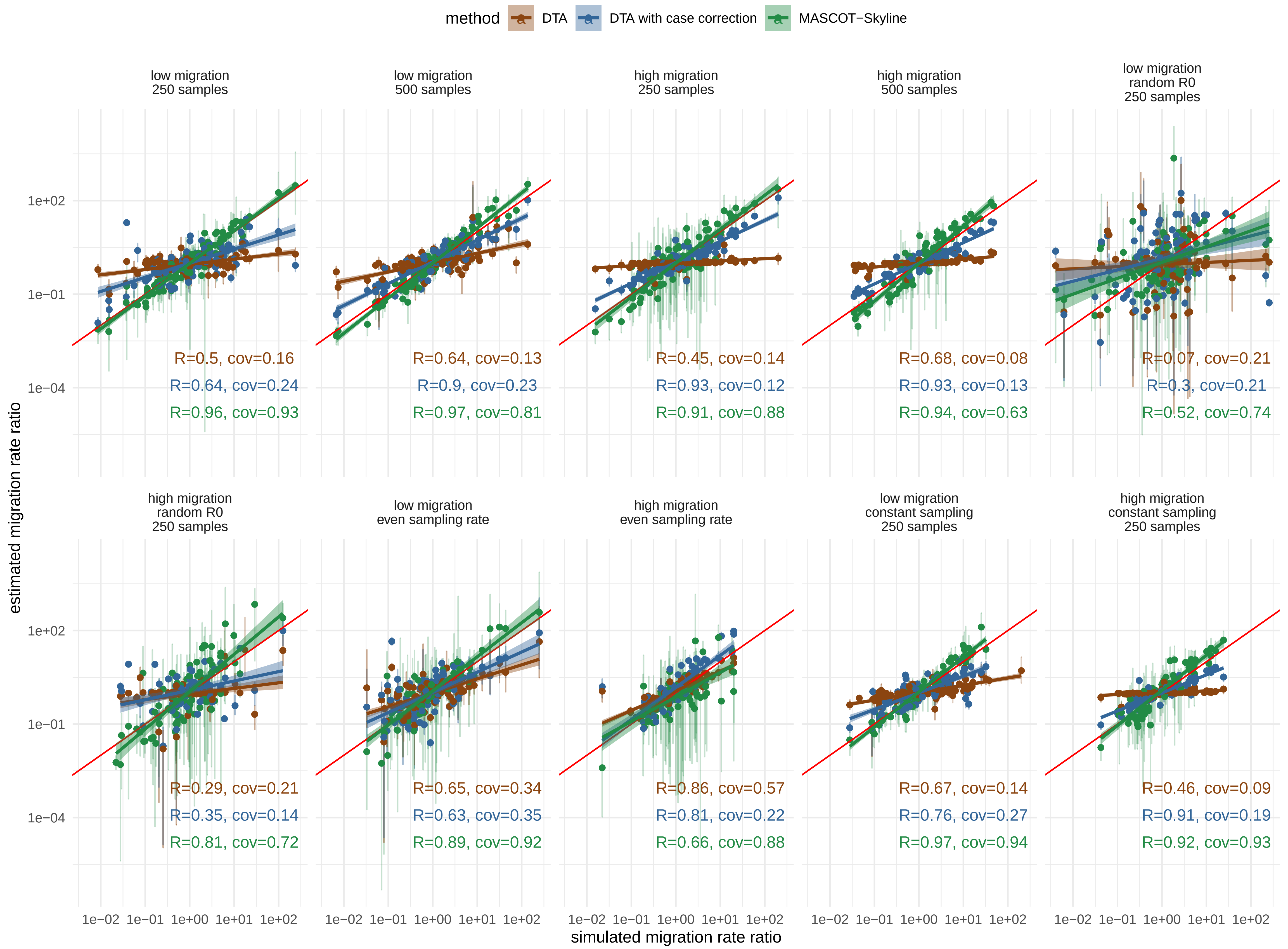

method
DTA
DTA with case correction
MASCOT−Skyline
low migration 250 samples
low migration 500 samples
high migration 250 samples
high migration 500 samples
low migration random R0 250 samples
R=0.5, cov=0.16
R=0.64, cov=0.24
R=0.96, cov=0.93
R=0.64, cov=0.13
R=0.9, cov=0.23
R=0.97, cov=0.81
R=0.45, cov=0.14
R=0.93, cov=0.12
R=0.91, cov=0.88
R=0.68, cov=0.08
R=0.93, cov=0.13
R=0.94, cov=0.63
R=0.07, cov=0.21
R=0.3, cov=0.21
R=0.52, cov=0.74
high migration random R0 250 samples
low migration even sampling rate
high migration even sampling rate
low migration constant sampling 250 samples
high migration constant sampling 250 samples
R=0.29, cov=0.21
R=0.35, cov=0.14
R=0.81, cov=0.72
R=0.65, cov=0.34
R=0.63, cov=0.35
R=0.89, cov=0.92
R=0.86, cov=0.57
R=0.81, cov=0.22
R=0.66, cov=0.88
R=0.67, cov=0.14
R=0.76, cov=0.27
R=0.97, cov=0.94
R=0.46, cov=0.09
R=0.91, cov=0.19
R=0.92, cov=0.93
estimated migration rate ratio
1e+02
1e−01
1e−04
simulated migration rate ratio
1e−02 1e−01 1e+00 1e+01 1e+02
