# Supplementary material for: MASCOT-Skyline integrates population and migration dynamics to enhance phylogeographic reconstructions
Source: PLoS Comput Biol. 2025 Sep 26;21(9):e1013421. doi: 10.1371/journal.pcbi.1013421 (PMC12500135; doi:10.1371/journal.pcbi.1013421)
Supplement: S21 Fig — Here, we compare the computational speed of MASCOT-Skyline and MASCOT-Constant for different-sized datasets that are not sampled through time. As a result, the maximum number of co-existing sequences is the number of samples. We then inferred the population dynamics from the true phylogenetic trees using MASCOT-Skyline and MASCOT-Constant. Next, we plot the ESS per hour of the posterior probability of MASCOT-Skyline over the ESS per hour of the MASCOT-Constant for inference from the same phylogenetic tree (y-axis). The x-axis shows the total number of samples or leaves in the simulation. The different colors show the number of demes in the simulations. D ESS per hour (skyline model, log-scale) vs. number of samples. F ESS per hour (constant model, log-scale) vs. number of samples. (PDF) [file pcbi.1013421.s021.pdf]

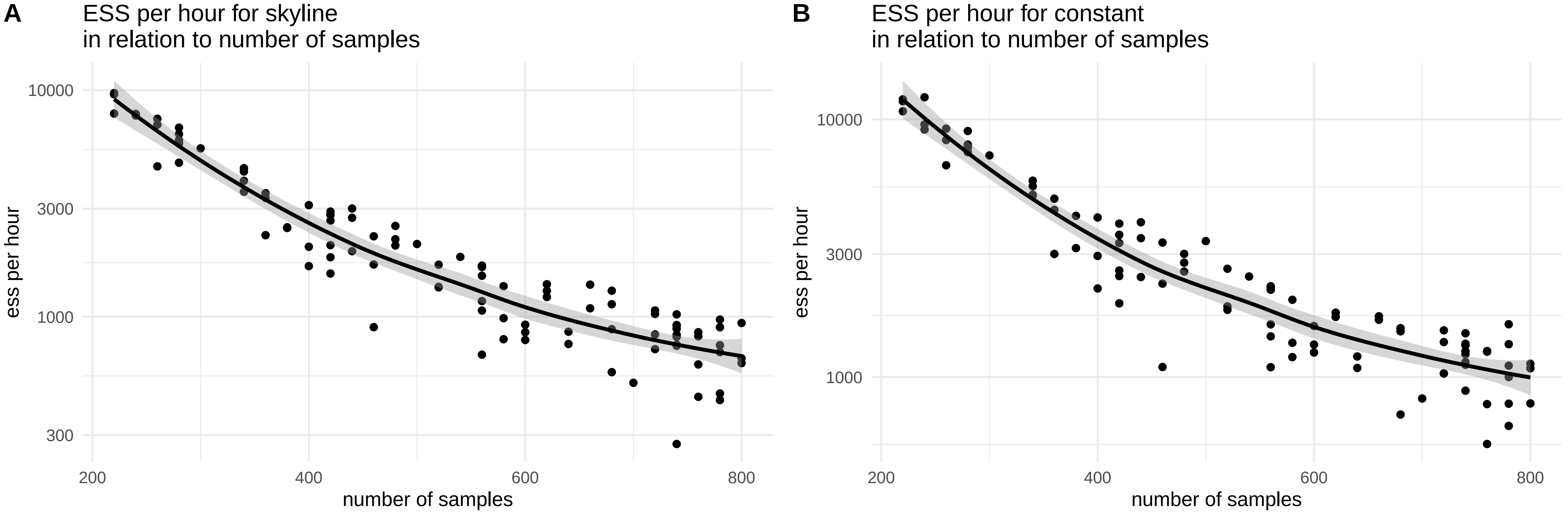
A
ESS per hour for skyline
in relation to number of samples
10000
3000
1000
300
ess per hour
200
400
600
800
number of samples
B
ESS per hour for constant
in relation to number of samples
10000
3000
1000
ess per hour
200
400
600
800
number of samples
