# Supplementary material for: MASCOT-Skyline integrates population and migration dynamics to enhance phylogeographic reconstructions
Source: PLoS Comput Biol. 2025 Sep 26;21(9):e1013421. doi: 10.1371/journal.pcbi.1013421 (PMC12500135; doi:10.1371/journal.pcbi.1013421)
Supplement: S1 Text — (PDF) [file pcbi.1013421.s024.pdf]

SUPPLEMENTAL TABLE

Data Availability

GISAID Identifier:	EPI_SET_240311hz
doi:	10.55876/gis8.240311hz

All genome sequences and associated metadata in this dataset are published in GISAID's EpiCoV database. To view the contributors of each individual sequence with details such as accession number, Virus name, Collection date, Originating Lab and Submitting Lab and the list of Authors, visit 10.55876/gis8.240311hz

Data Snapshot

- EPI_SET_240311hz is composed of 13,866 individual genome sequences.
- The collection dates range from 2019-12-26 to 2020-11-10;
- Data were collected in 127 countries and territories;
- All sequences in this dataset are compared relative to hCoV-19/Wuhan/WIV04/2019 (WIV04), the official reference sequence employed by GISAID (EPI_ISL_402124). Learn more at https://gisaid.org/WIV04.
